# Plasma-Activated Medium Potentiates the Immunogenicity of Tumor Cell Lysates for Dendritic Cell-Based Cancer Vaccines

**DOI:** 10.3390/cancers13071626

**Published:** 2021-04-01

**Authors:** Sergej Tomić, Anđelija Petrović, Nevena Puač, Nikola Škoro, Marina Bekić, Zoran Lj. Petrović, Miodrag Čolić

**Affiliations:** 1 Department for Immunology and Immunoparasitology, Institute for the Application of Nuclear Energy, University of Belgrade, 11080 Belgrade, Serbia; marina.bekic@inep.co.rs (M.B.); miocolic@gmail.com (M.Č.); 2 Institute of Physics, University of Belgrade, 11080 Belgrade, Serbia; andjelija@ipb.ac.rs (A.P.); nskoro@ipb.ac.rs (N.Š.); 3 Serbian Academy for Sciences and Arts, 11000 Belgrade, Serbia; zoran@ipb.ac.rs; 4 School of Engineering, Ulster University, Jordanstown, Co. Antrim BT37 0QB, UK; 5 Medical Faculty Foca, University of East Sarajevo, 73 300 Foča, Bosnia and Herzegovina

**Keywords:** plasma activated medium, dendritic cells, tumor vaccines, Th polarization

## Abstract

**Simple Summary:**

Dendritic cells (DCs)-based anti-cancer vaccines displayed limited efficacy in clinical trials, mostly due to a lack of protocols for preparing immunogenic tumor antigens used in the vaccine. Here, a unique atmospheric pressure plasma jet was used to prepare a plasma-activated medium (PAM) which induced immunogenic cell death in tumor cells. This procedure increased the efficacy of tumor lysates in enhancing the immunogenicity of DCs according to their increased maturation, production of IL-12, and the capacity to induce cytotoxic CD8 T cells able to kill tumor cells. In contrast to the tumor lysates commonly used in DC vaccines, PAM-tumor lysates lacked the capacity to increase IL-10 production by DCs, and their potential to induce protumorogenic Th2 and regulatory T cells. Cumulatively, these results suggest that the novel method for preparing immunogenic tumor lysates with PAM could be suitable for improved DC-based immunotherapy of cancer patients.

**Abstract:**

Autologous dendritic cells (DCs)-based vaccines are considered quite promising for cancer immunotherapy due to their exquisite potential to induce tumor antigen-specific cytotoxic T cells. However, a lack of efficient protocols for inducing immunogenic tumor antigens limits the efficacy of DC-based cancer vaccines. Here, we found that a plasma-activated medium (PAM) induces immunogenic cell death (ICD) in tumor cells but not in an immortalized L929 cell line or human peripheral blood mononuclear cells. PAM induced an accumulation of reactive oxygen species (ROS), autophagy, apoptosis, and necrosis in a concentration-dependent manner. The tumor lysates prepared after PAM treatment displayed increased immunogenicity in a model of human monocyte-derived DCs, compared to the lysates prepared by a standard freezing/thawing method. Mature DCs loaded with PAM lysates showed an increased maturation potential, as estimated by their increased expression of CD83, CD86, CD40, IL-12/IL-10 production, and attenuated PDL1 and ILT-4 expression, compared to the DCs treated with control tumor lysates. Moreover, in co-culture with allogeneic T cells, DCs loaded with PAM-lysates increased the proportion of cytotoxic IFN-γ+ granzyme A+ CD8+ T cells and IL-17A-producing T cells and preserved the Th1 response. In contrast, control tumor lysates-treated DCs increased the frequency of Th2 (CD4+IL-4+), CD4, and CD8 regulatory T cell subtypes, none of which was observed with DCs loaded with PAM-lysates. Cumulatively, these results suggest that the novel method for preparing immunogenic tumor lysates with PAM could be suitable for improved DC-based immunotherapy of cancer patients.

## 1. Introduction

Cold atmospheric plasma (CAP), also called non-equilibrium atmospheric-pressure plasma (NEAPP), is a partially ionized gas generated under normal atmospheric pressure and ambient temperature [1]. Achieving non-equilibrium conditions at atmospheric pressure proved to be possible in only a limited number of cases. Just recently, CAP sources have become more diversified and are being developed with specific applications in mind. CAP is a source of reactive species, ions, neutral particles and molecules, electrons, and other physical factors such as electromagnetic fields, metastable and excited molecules, weak ultraviolet radiation, etc., while producing only a very weak or negligible heating effect [2]. CAP has been widely used in various fields of modern medicine, such as promoting wound healing, blood coagulation, stem cell differentiation and the treatment of some skin diseases, as an anti-bacterial, anti-viral and sterilization agent [3]. In particular, CAP has recently been tested for cancer treatment [2,4,5,6]. The therapeutic effect of low-temperature plasma is based on the production of various reactive oxygen and nitrogen species (RONS) [7], such as nitric oxide (•NO) and hydroxyl (•OH) radicals [8]. In contact with the cancer tissue, CAP produced RONS are capable of inducing cell death and this approach has been extensively explored for the treatment of tumor because malignant cells are extremely vulnerable to the effect of RONS and die by necrosis, apoptosis, or necroptosis. In contrast, normal cells are less sensitive to CAP [9]. An indirect approach to using CAP in medicine is the production of a plasma-activated medium (PAM). The gas phase plasma chemistry and the plasma chemistry in the gas/liquid (i.e., cell culture medium) interface induce chemical reactions in the liquid phase. As a result, specific compounds are created in the liquid phase which are responsible for the effects on cells (bacteria, plant cells, human cells, cancer cells, stem cells, etc.). The chemistry, both in a gas phase and liquid phase, depends on the plasma parameters (type of gas mixture, gas flow, concentration of electrons, temperature of electrons, deposited power etc.), and on the type of liquid medium that is being treated by plasma. PAM may be prepared by treating aqueous solutions, including cell culture media, with CAP. In this process, RONS are transported from the gas phase into the liquid surface, dissolved into the medium, and undergo further reactions with dissolved molecules in the aqueous solution. Mixing of the gas-phase RONS with the medium is promoted (in this paper as well) by a strong flow of gas/plasma into the liquid whereby “bubbles” of plasma effluent are formed within the top layer of the liquid. 

The interaction of gas-phase RONS with aqueous organics produces other relatively long-lived RONS, such as hydrogen peroxide (H2O2), nitrates, nitrites and organic peroxides (RO2) [8]. Due to the long-lived RONS, PAM has been shown to be as effective in killing cancer cells as direct treatment with CAP, and the effect of both treatments is enhanced by intracellular ROS production [10]. The “treatment dose” depends on the source of the plasma, the time of plasma exposure of the liquid, as well as the period for which the cells or tissue is allowed to remain in contact with PAM [11]. The efficacy of plasma in the treatment of malignant tumors is based on two general principles. The first includes a direct cytotoxic effect caused by RONS- mediated intracellular oxidative stress followed by inactivation by anti-oxidative mechanisms. This phenomenon is of special relevance, knowing that the increase of pro-oxidative mechanisms in the tumor could be beneficial for tumor therapy [12]. The second pathway involves activation of the anti-tumor immune response by molecules released from CAP- and PAM-dependent immunogenic cell death (ICD) [13].

ICD is a type of cell death, also referred to as immunogenic apoptosis, which is characterized by a release or display of damage-associated molecular patterns (DAMPs). The most important DAMPs released from cancer cells during ICD are calreticulin, adenosine triphosphate (ATP), heat-shock proteins, and high mobility group protein B1 (HMGB1). DAMPs are a potent adjuvant for antigen-presenting cells (APCs), especially dendritic cells (DCs), inducing their migration to the regional lymphoid tissue, maturation, and stimulation of a specific anti-tumor immune response [14]. DCs are specialized APCs playing a key role in the initiation and regulation of innate and adaptive immune responses. Due to their unique properties, DCs have been extensively investigated and used to improve cancer immunotherapy. In this context, many strategies have been developed to target DCs in cancer [15]. One of them is the in vitro generation of DCs from monocytes (MoDCs), loading of these APCs with autologous tumor lysates together with maturation stimuli, and inoculation of thus prepared DCs vaccines in cancer patients [16]. The use of DC vaccines for cancer therapy has been extensively investigated, with more than 200 completed clinical trials to date. The injected MoDCs migrate to the regional lymph nodes where they efficiently induce CD4+ T cell- and CD8+ T cell-mediated anti-tumor responses. MoDCs can be also recruited into the tumor microenvironment (TME) following treatment with ICD inducers, including CAP, with the potency to the prime anti-tumor T-cell responses [15]. The clinical benefit of DC-based vaccines is not as efficient as expected due to the presence of many inhibitory molecules and mechanisms in TME. Therefore, an immunotherapy approach based on improved DC vaccines together with the blockade of inhibitory molecules in TME sounds promising for future tumor therapy [15,17]. Optimization of DC vaccines implies the use of better protocols for preparing tumor antigens, designing new strategies for antigen loading, as well as selection of optimal adjuvants and maturation stimuli. In this context, the use of lysates from tumor cells subjected to ICD could be beneficial, as already well documented [14,18,19,20]. PAM treatment of tumor cells has not been explored up to the date for the stimulation of MoDCs. This was the reason why we tested the hypothesis that a lysate prepared from PAM-treated tumor cells is superior to the induction of immunogenic DCs than the tumor lysates prepared under the conventional freezing-thawing protocol, commonly employed in clinics.

## 2. Materials and Methods

### 2.1. Experimental Setup-Plasma Treatments 

**Figure 1:**
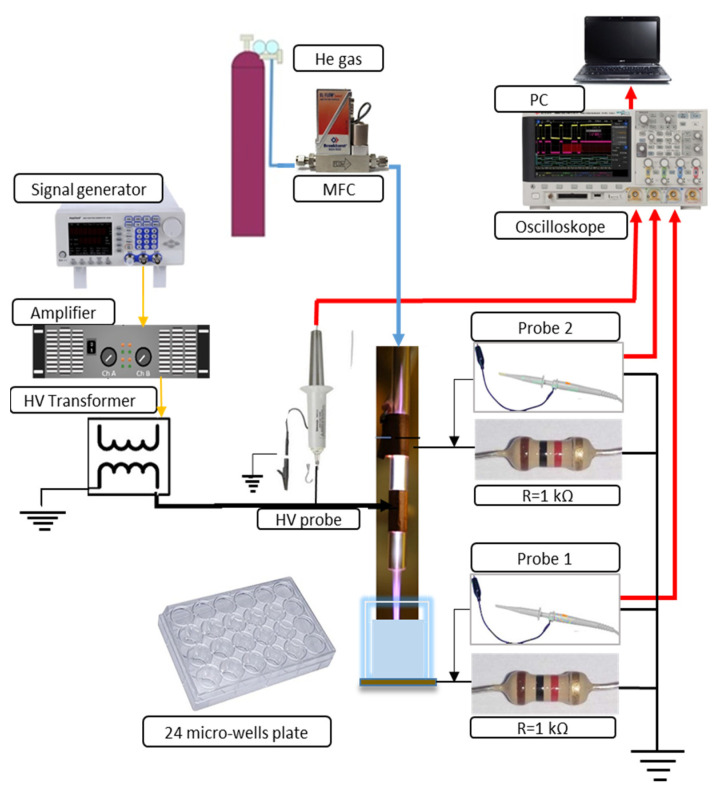
Schematic diagram of the experimental setup.

Figure 1 illustrates the schematics of the dielectric barrier discharge (DBD) atmospheric pressure plasma jet (APPJ) setup and the plasma treatment of a medium placed in the well of a 24-well plate. The DBD jet consists of a glass tube with an inner diameter of 4 mm and an outer diameter of 6 mm. The two electrodes placed around the tube are 15 mm wide and made of copper tape. The surface of the target grounded through a resistor served as the third electrode. The electrode positioned at 5 mm from the edge of the glass tube was connected to the power supply. Another grounded electrode was placed at a distance of 10 mm from the powered electrode. This electrode was grounded over a second resistor to the same point of the electrical circuit as the ground line of the target. The power supply of the APPJ consists of a function generator (PeakTech DDS Function Generator, PeakTech GmbH, Ahrensburg, Germany), a home-made amplifier, and a high-voltage transformer operating at its resonance frequency of 81 kHz. A sine-wave high-voltage signal was provided to power the APPJ. One high-voltage (HV) probe (P6015A HV probe, Tektronix Inc., Beaverton, OR, USA) and two voltage probes (N2863B voltage probe, Agilent, Santa Clara, CA, USA) were used to record voltage and current waveforms. The current waveforms were obtained at the resistors (R = 1kΩ) in the grounded branches of the electrical circuit. They were used to monitor the stability of plasma and calculate the power delivered from the plasma to the sample. This power was 1.2 W in all experiments. The RMS voltage was 1.9 kV and the RMS current was 2.2 mA. The APJ was operating with 2 slm of He as the working gas. The distance between the ending of the APPJ tube and the surface of the sample was 5 mm in all experiments. After the sample treatment, the production of RONS in PAM was investigated by spectrophotometry and colorimetric methods and the concentrations of nitrite ions, nitrate ions and hydrogen peroxide were determined. The pH value of the PAM-RPMI 1640 was not changed after the plasma treatment.

### 2.2. Measurement of RONS in the Plasma-Activated Medim

Immediately after the plasma treatment PAM was placed in vials and frozen in liquid N_2_. RONS (H_2_O_2_, NO_2_^−^, NO_3_^−^) were measured by using colorimetric measurements. The measurements were performed immediately after the plasma treatment and, as a control, after defrosting the PAM. No differences in RONS concentrations were noticed between these samples (data not shown). This is in accordance with the previously reported results. The good stability of PAM properties in room conditions has been shown to be preserved for 8 to 18 h [21]. Moreover, freeze-thaw procedures for different PAM were studied and it reportedly retains the properties when kept at low temperatures [22].

The H_2_O_2_ and NO_3_^-^ concentrations of the treated medium were analyzed by using commercial H_2_O_2_ test stripes (Merckoquant 110011, Merck, Darmstadt, Germany) and commercial NO_3_^−^ test stripes (Merckoquant 110020, Merck, Darmstadt, Germany). The test stripes were scanned by a scanner (Perfection V370 Photo, Epson, Nagano, Japan) in order to analyze their color value. A calibration procedure was performed before the experiments. Six different H_2_O_2_ concentrations ranging from 0 to 25 mg/L were made by diluting 30% H_2_O_2_ stock solution in distilled H_2_O. The same process of calibration was used for the nitrate concentration. Seven different nitrate concentrations ranging from 0 to 250 mg/L were made by diluting a nitrate stock solution (Nitrate standard solution 200 mg/L NO_3_-N in H_2_O, 125040, Merck, Darmstadt, Germany) in distilled H_2_O. The sum of the color values of the red, green, and blue channel (grey value) for different H_2_O_2_ and NO_3_^−^ concentrations were plotted in their respective calibration graphs. The exponential fit of the calibration data allows the determination of an unknown H_2_O_2_ and NO_3_^−^ concentration deposited in the sample during the plasma treatment. Aliquots of 10 µL PAM were taken immediately after 5 min of plasma treatment time. The plasma treatment was done in triplicate and each time concentration of H_2_O_2_ and NO_3_^−^ was determined.

To detect nitrite ions (NO_2_^−^), Griess reagent was added to the PAM (Spectroquant Nitrite test 1.14776.0001, Merck, Darmstadt, Germany). In an acidic solution, nitrite ions react with sulfanilic acid to form a diazonium salt, which in turn reacts with N-(1-naphthyl) ethylenediamine dihydrochloride to form a red-violet azo dye. This dye was determined spectrophotometrically and the absorbance was measured at λ = 540 nm. For the calibration curve, seven different nitrite concentrations ranging from 0 to 1 mg/L NO_2_-N were produced by diluting a nitrite stock solution (Nitrate standard solution 40 mg/L NO_2_-N in H_2_O, 125042, Merck, Darmstadt, Germany) in distilled water. Similarly to NO_3_^−^ and H_2_O_2,_ the concentration of NO_2_^−^ was determined after 5 min plasma treatments. The experiment was done in triplicate.

### 2.3. Cells 

Human melanoma A375, laryngeal carcinoma Hep2, and immortalized mouse fibroblast L929 cell lines were obtained from ATCC (American Type Cell Culture, Manassas, WV, USA) and were stored in 10% DMSO/Fetal calf serum (FCS, Sigma, St. Louis, MO, USA) in liquid nitrogen before the experiments. The cells were thawed in 20x volume of complete RPMI medium, containing basal RPMI 1640, 10% FSC, 2 mM L-glutamine (Sigma, St. Louis, MO, USA), 50 µM 2-mercaptoethanol (2-ME, Sigma, St. Louis, MO, USA ), and antibiotics (gentamicin, streptomycin, penicillin) and after washing by centrifugation, they were cultivated at 37 °C, 5% CO_2_ up to 80% confluence. After reaching the confluence, the cells were passaged via trypsinization in 0.2% trypsin/0.02% NaEDTA (Sigma, St. Louis, MO, USA) according to standard laboratory procedures. 

Peripheral blood mononuclear cells (PBMCs) were obtained from healthy volunteers who signed consent forms and isolated by using Lymphoprep (Nycomed, Oslo, Norway). All studies involving the usage of human cells were approved by the Ethics committee of the Institute for the Application of Nuclear Energy, University of Belgrade (No. 02/765/2). PBMC were used in direct cytotoxicity assay or as a source for the isolation of monocytes and T cells as negative fractions of Magnetic-activated cell sorting (MACS) using Monocyte isolation kit II and Pan-T cell isolation kits (Miltenyi Biotec, Bergisch Gladbach, Germany), respectively. 

MACS-sorted monocytes were used for the generation of monocyte-derived dendritic cells (DCs) according to a protocol described previously [23]. Briefly, 1 × 10^6^ monocytes were plated in low-adherent 6-well plates (Sarstedt AG & Company, Sarstedt, Germany) and cultivated for 4 days in CellGenix^®^ GMP DC medium (CellGenix, Freiburg im Breisgau, Germany) in the presence of a recombinant human granulocyte-macrophage colony-stimulating factor (GM-CSF) and interleukin (IL)-4 (both at 20 ng/mL, R & D Systems, Minneapolis, MN, USA) to obtain immature (im)DCs. Tumor lysates derived from A375 or Hep2 cells, as described in Section 2.4, were added to the DC cultures on day 4. To obtain mature (m)DC, the cells were treated with LPS from *E. coli* 0.111:B4 (200 ng/mL, Sigma, St. Louis, MO, USA) and human recombinant IFN-γ (20 ng/mL), 4 h after the treatment of DCs with tumor lysates, for the next 16–18 h. Afterward, the DCs were collected and used for phenotype characterization and functional assays with MACS-purified T cells. 

### 2.4. Cytotoxicity Study

PAM was prepared by treating a basic RPMI 1640 medium with CAP. The components for a complete RPMI medium were added immediately afterwards and serial dilutions of PAM were prepared in the complete RPMI medium. 

To evaluate the cytotoxic effects of PAM on A375, Hep2, L929 cells (each at 5 × 10^4^ cells per well of a flat 96-well plate) and PBMCs (2 × 10^5^ cells per well of 96-wells plate) were cultivated in complete RPMI medium with serial dilutions of PAM (100%, 50%, 25%, 12.5%, 6.25%, 0%) for 24 h. Supernatants of these cell-cultures were collected and stored at −80 °C for cytokines analysis. The MTT assay was performed on the remaining cells to determine the metabolic activity of the cells treated with PAM. The corresponding cell-free cultures containing PAM were used as blank controls. After the cultivation, all cultures were washed in phenol red-free RPMI medium twice and MTT (3-(4,5-dimethylthiazol-2-yl)-2,5- diphenyltetrazolium bromide (1 mg/mL), Sigma) in phenol red-free RPMI was added for the next 4 h. The formazan crystals were dissolved by using 10% sodium dodecyl sulphate (SDS, Sigma, St. Louis, MO, USA) in 0.01N HCl (Sigma, St. Louis, MO, USA) overnight, and the absorbance was read at 570 nm with a spectrophotometer (ELx800, Biotek, Winooski, VT, USA). The absorbance in cell-free blank controls was subtracted from the absorbance of corresponding experimental cultures. Each of the three experiments performed was carried out in six-plicates. The metabolic activity (MTT%) detected in the treated cultures was expressed as the percentage of the absorbance in non-treated control cultures (100%). 

Oxidative stress in the A375 cells treated with different concentrations of PAM (6.25–100%) for 4 h or 24 h was analyzed after trypsinization of cells, by using a Muse^®^ Oxidative Stress Kit (Luminex, Austin, TX, USA), which is based on the reactive oxygen species-sensitive dye dihydroethidium (DHE), according to manufacturer’s instruction. Autophagy flux in the A375 cells treated with different concentrations of PAM was evaluated using a Muse ^®^ Autophagy LC3 Antibody-based kit, according to the manufacturer’s instructions. The method relies on the detection of membrane-bound LC3 after selective permeabilization of cells which extracts cytoplasmic LC3 but not membrane-bound LC3, and the blockage of lysosomal degradation of LC3 in autophagosomes. Apoptosis of the A375 cells treated with PAM for 24 h was detected with a Muse ^®^ Annexin V & Dead Cell kit, which is based on Annexin V/7AAD staining in Ca^2+^-containing buffer. The analysis of oxidative stress, autophagy, and apoptosis was performed on a Guava^®^ Muse^®^ Cell Analyzer (Luminex, Austin, TX, USA). The expression of heat-shock protein (HSP) 60 and heat-shock complex (HSC) 70 on the surface of A375 and Hep2 cells was analyzed after 4h exposure of cells to different doses of PAM. After harvesting the cells by trypsinization, the cells were incubated with primary mouse anti-HSP 60 (Clone 24, 1 µg/mL BD Biosciences, San Jose, CA, USA) or mouse anti-HSC 70 (Clone sc-7298, 1 µg/mL Santa Cruz Biotechnology, Inc., Dallas, TX, USA) in PBS/NaN3/5%FCS for 30 min, washed, and then incubated with a secondary anti-mouse IgG-FITC antibody (Sigma St. Louis, MO, USA) for 20 min, followed by fixation and analysis by flow cytometry (BD LSR II).

A375 and Hep2 tumor cells treated with PAM for 24 h, and control non-treated tumor cells, were used for preparing tumor lysates in DC cultures. Totally 5 × 10^6^ live tumor cells were incubated in 500 µL of 100% PAM for 24 h, and a loss of viability >90% in the PAM-treated cultures was confirmed by Trypan blue staining. Both PAM-treated and control tumor cells were then frozen at −80 °C for 10 min and afterward thawed at room temperature for 10 min in an ultrasonic bath, and the process was repeated at least 7 times. After that, 100 µL of complete tumor lysates were added in 2 mL of DC cultures (total 5% vol.) thus providing an equivalent of 1:1 DC: lysed tumor cells, respectively. 

Direct cytotoxicity of PAM on DCs was evaluated by treating 4-day DC cultures with 12.5% or 25% of PAM, whereas control DC cultures were treated with equivalent volumes of basal RPMI medium, for the next 24 h. Apoptosis was detected by an Annexin V-FITC/PI staining kit according to manufacturer’s recommendations (Thermo Fisher, Waltham, MA, USA) and analyzed on a flow cytometer (LSR II, Becton Dickinson, East Rutherford, NJ, USA). Oxidative status in the DCs treated with PAM for 24 h was assessed by staining the cells with 2.5 µM dihydrorhodamine (DHR) in PBS for 30 min at 37 °C, followed by analysis on the flow cytometer.

### 2.5. Mixed Cell Reactions

The allostimulatory capacity of DCs (prepared as in Section 2.3) was tested by co-cultivating MACS-purified T cells (1 × 10^5^/well of 96-well U-bottom plate) labeled with CellTrace™ Far Red (1 µM, Thermo Fisher, Waltham, MA, USA), with a different number of DCs (1 × 10^4^, 0.5 × 10^4^, 0.25 × 10^4^ cells/well) for 5 days. To avoid transferring any stimuli from DC cultures, the cells were washed twice in RPMI medium prior to co-cultivation with T cells. After 5-day co-cultures, the cells were washed in PBS, stained with 7AAD and then analyzed on a BD LSR II flow cytometer. CellTrace Far Red dilution was analyzed after exclusion of doublets and dead (7AAD+) cells, and the percentage of proliferated T cells (CellTrace Far Red ^low^) was calculated in FCS Express (DeNovo Software, Pasadena, CA, USA). The capacity of DCs to induce allogeneic cytokines production by T cells was analyzed after 5-day co-cultures (1:20 DC:T cell ratio), after treating the co-cultures with PMA (20 ng/mL, Sigma, St. Louis, MO, USA), calcium ionophore (500 µg/mL, Sigma, St. Louis, MO, USA) and monensin (3 µg/mL, Sigma, St. Louis, MO, USA) for the last 4 h to stimulate cytokine accumulation in the primed T cells. Harvested cells were washed in 0.1% NaN3/PBS and stained for surface and intracellular cell markers. To detect cytokines produced in co-culture-supernatants, DC/T cell co-cultures carried out likewise, were treated with PMA and Ca Ionophore for the last 4 h, and the supernatants were collected after centrifugation at 2000 RPM for 5 min. 

To evaluate the capacity of DCs to prime cytotoxic T cells, autologous T cells isolated from freshly obtained PBMC were co-cultured with A375 lysate-loaded or non-loaded DCs at a 1:40 DC:T-cell ratio for 6 days. Recombinant IL-2 (10 ng/mL) was added to these co-cultures on day 0 and day 3. After 6 days, the proliferation of autologous T cells was evaluated by staining the cells fixed in ice-cold ethanol (75%) for 2h at −20 °C and washed with PBS, with an anti-human Ki-67 antibody (Wuhan Fine Biotec Co., Wuhan, China) and secondary anti-rabbit IgG Alexa 647 (Abcam, Cambridge, UK), followed by staining with PI (1 ug/mL, Sigma, St. Louis, MO, USA) prior to the analysis of proliferation on a flow cytometer. Cytotoxic activity of the primed autologous T cells towards live A375 tumor cells was carried out by co-cultivating T cells with CellTrace Far Red-labeled live A375 cells (5 × 10^5^ cells) at 1:2, 1:4 and 1:8 A375: T cell ratios for 4 h. After that, the cells were collected and labeled with PI prior to analysis on a flow cytometer. For all mixed cell cultures, T cells cultivated without DCs and A375 cells cultivated without T cells were used as controls. 

### 2.6. Flow Cytometry

The flow cytometry analysis of DCs and T cells was performed on the flow cytometers CyFlow Cube 6 (Sysmex, Kobe, Hyogo, Japan) and LSR II (BD) by staining the cells with the following directly conjugated antibodies: anti-CD83-FITC, anti-CD86-PE, CD86-PerCPCy5.5, anti-CD40-APC, anti-CCR7-FITC, anti-IL17A-Alexa Fluor 488, anti-CD25-PerCPcy5.5, anti-CD127-PE, anti-IL10-APC, anti-HLA-DR-APCCy7 (Biolegend Inc., San Diego, CA, USA), anti-HLA-DR-PerCP (Miltenyi Biotec, Bergisch Gladbach, Germany), anti-ILT4-PE, anti-IFNγ-FITC (R&D Systems Minneapolis, MN, USA), anti-IL-12p40/p70-PE, anti-IL-10-FITC (BioRad, Hercules, CA, USA), anti-CD4-APC, anti-CD8-PerCPCy5.5, anti-Granzyme A-PE (eBioscience, San Diego, CA, USA), anti-Foxp3-FITC, anti-IL-4-PerCP, anti-PDL1-PE (eBioscience, San Diego, CA, USA), anti-CD4-PE (Sysmex, Kobe, Hyogo, Japan), IgG1 negative control-PE, IgG1 negative control-FITC, IgG1 negative control-APC, IgG1 negative control PerCP, (Thermo Fisher, Waltham, MA, USA), IgG2 negative control APCCy7 (Millipore, Burlington, MA, USA). Surface staining with primary Abs was conducted in PBS/0.1% NaN3/0.5% FBS prior to the intracellular staining that was carried out using a flow cytometry fixation and permeabilization kit (Biolegend, San Diego, CA, USA). Signal overlap between the channels was compensated before each analysis using single labeled samples. Non-specific fluorescence was determined according to isotype control antibodies and fluorescence minus one (FMO) controls and at least 5000 cells were analyzed in each sample. Doublets were excluded according to forward scatter (FS) H/FSA, and dead cells were gated-out according to 7-aminoactinomycin D (7AAD) staining, fixable viability dye 620 (BD) staining, or low FSC properties.

### 2.7. Cytokines 

TNF-α, IL-1β, IL-6, TGF-β, IL12-p70, IL-10, IL-23 and IL-27 were measured in DC culture supernatants by a specific duo-set sandwich enzyme-linked immunosorbent assay (ELISA) (R&D Systems, Minneapolis, MN, USA) in duplicates according to manufacturer’s protocol. The levels of cytokines in DC/T-cell co-cultures were determined by the LegendPlex human Th cytokine panel 13-plex (Biolegend) in duplicates, according to the manufacturer’s protocol. Unknown concentrations of cytokines were calculated from standard curves generated with the manufacturer-supplied recombinant cytokines and fitting with a 4-parameter (log) dose/response curve (GraphPad Prism 8, GraphPad Software, San Diego, CA, USA).

### 2.8. Statistical Analysis

Repeated-measures one-way analysis of variance (RM-ANOVA) was performed, followed by Tukey’s multiple comparison test, to analyze differences in means between the different groups of treated cells and control groups (GraphPad Prism 8,). Student’s T test was used to evaluate differences in levels of RONS in PAM. Data are presented as means ± SD of the indicated number of independent experiments (different time for experiments with cell lines, different DC donors and/or DC/T cell pairs), and differences were considered significant at *p* values of ≤0.05. For data presented as a heatmap, the values of each cytokine obtained in each experiment were normalized to the range 0–1, according to the following formula:

(1) $$y=\bar{X(}\frac{x-\min\left(x\right)}{\max\left(x\right)-\min\left(x\right)})$$

with y- heatmap value; x- level of cytokine in a sample in one dataset, (x)-dataset of one type of cytokine

## 3. Results and Discussion

### 3.1. Two-Electrode Plasma Jet Induces Efficient Accumulation of RONS in PAM

**Figure 2:**
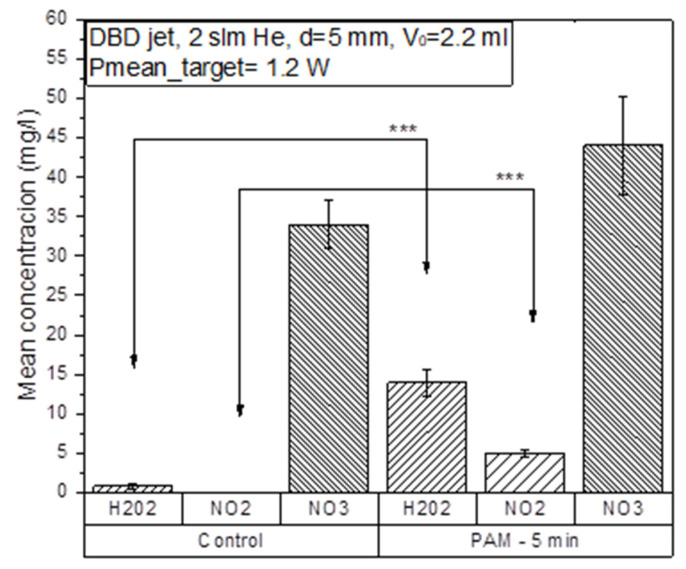
Measured concentrations of RONS in the plasma-activated medium PAM- RPMI 1640 compared to concentrations in the untreated RPMI 1640 medium. Helium flow during the treatments was kept constant at 2 slm and the power deposited in the discharge in contact with the sample was 1.2 W. The distance between the edge of the APPJ and the sample surface was 5 mm in all treatments. *** *p* < 0.005 compared to non-treated control RPMI 1640 (0% PAM) or as indicated by line.

A two-electrode dielectric barrier discharge (DBD) atmospheric pressure plasma jet (APPJ) was used as a CAP source (Figure 1) for treating the RPMI 1640 cell culture medium. The power deposited in the effluent discharge in contact with the surface of RPMI 1640 was 1.2 W. The resistors (R = 1 kΩ) in this grounded branch of the electrical circuit used for monitoring current waveforms showed stable plasma throughout the PAM generation. The distance of the APPJ to the treated liquid was kept constant at 5 mm in all experiments and treatment time was 5 min. Plasma treatment did not change the pH of the medium, according to stable phenol-red coloring and the measurements of pH. We have measured the three long-lived RONS and their concentrations in untreated RPMI 1640 and the PAM-RPMI 1640 are shown in Figure 2. In the literature, H_2_O_2_ is the most commonly detected and characterized, followed by NO_2_^–^. At the end of the list is the NO_3_^–^ radical, which is scarcely reported albeit it plays an important role in PAM-cell interactions. Their concentration mainly depends on the type of plasma device used, deposited power in the discharge, feeding gas, treatment time, and treated volume. Adding the type of the treated liquid media to this large variety of parameters that can influence the concentration of deposited RONS in the cell medium makes it very difficult to perform a direct comparison of two atmospheric plasma systems [24]. Nevertheless, the described diagnostics provide enough information to ascertain the stability of plasma and identify the most abundant species.

The measured concentration of hydrogen peroxide was 1 mg/L and of nitrate 34 mg/L, while there was no nitrite detected in the untreated RPMI 1640 medium. The largest increase in concentration was observed for hydrogen peroxide, which increased 14 times after the treatment. Hydrogen peroxide is relatively stable and has strong oxidizing properties that can cause lipid peroxidation and, among other roles, serves as a cellular messenger. In the case of PAM-RPMI 1640, the highest yield of H_2_O_2_ was expected due to the presence of organic molecules in the cell medium (like glucose) [25]. Regardless of some reports in the literature stating that the increase in the concentration of H_2_O_2_ is generally responsible for the decrease in the viability of cancer cells [22,26], the main reason for this is the synergistic effect with reactive nitrogen species [27]. Therefore, we monitored the concentration of nitrates and nitrites in PAM-RPMI 1640. The concentration of nitrites in PAM was 5 mg/L and the nitrate concentrations increased by 30% compared to the untreated control RPMI medium. 

### 3.2. PAM Induces Immunogenic Cell Death in Tumor Cells

**Figure 3:**
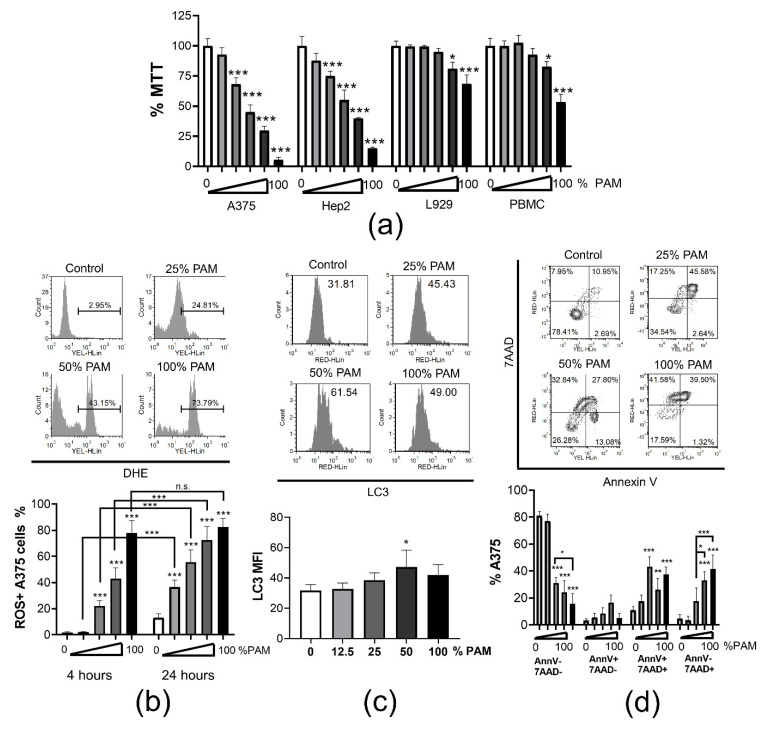
Dose dependent cytotoxicity of PAM. (**a**) Metabolic activity of A375, Hep2, L929, PBMC was assessed by MTT assay after 24 h treatment with serial dilutions of PAM (0% (white bars), 6%, 12.5%, 25%, 50% and 100% (black bars)) in complete RPMI medium. The results are shown as % MTT relative to the non-treated corresponding control cells 100%. Data from two independent experiments, each carried out in triplicates, are shown as the average % of MTT ± SD. (**b**) A representative data on the measurement of oxidative stress by DHE in A375 cells cultivated in the presence different concentrations of PAM for 4 h is shown. The summarized data from two independent experiments, in which the oxidative stress with the different doses of PAM (0% (white bars), 12.5%, 25%, 50% and 100% (black bars)) was measured after 4 h or 24 h as indicated, is shown as mean % of ROS+ cells ± SD. (**c**) A representative histograms on measurements of membranous LC3 expression in permeabilized A375 cells is shown with the indicated total mean fluorescence intensity (MFI), and the summarized data from two experiments is shown as MFI of LC3 ± SD. (**d**) Analysis of cell death by Annexin V/7AAD staining (AnnV−7AAD− viable; AnnV+7AAD− early apoptosis; AnnV+7AAD+ late apoptosis; AnnV−7AAD+ necrosis) in A375 cells is shown, carried out after 24 h cultures in presence of different doses of PAM (0%, 12.5%, 25%, 50%, 100%). Representative dot-plots and summarized data are shown as mean % A375 cells ± SD of 3 independent experiments. * *p* < 0.05, ** *p* < 0.01, *** *p* < 0.005 compared to non-treated control cells (0% PAM) or as indicated by line.

ICD of tumor was reported as beneficial for triggering a DC-mediated anti-tumor response [28]. Increased presence of RONS in PAM makes it a good candidate for inducing ICD in tumor cells. Therefore, we examined the dose-dependent cytotoxicity of PAM towards tumor A375 melanoma cell line and laryngeal carcinoma Hep2 cell line, as well as towards non-tumor cells, such as immortalized L929 cell line and PBMCs (Figure 3a). Results from an MTT assay suggested that even the low doses of PAM (12.5% and 25%) were cytotoxic for A375 and Hep2 cells after 24h culture, but not for L929 cells and PBMCs. Higher doses (50% and 100%) of PAM significantly reduced the metabolic activity of all cell types, but the effect was most prominent in cultures with the tumor A375 and Hep2 cells. According to the ISO standard on the cytotoxicity of medical devices, a 30% reduction in MTT is considered as non-cytotoxic [29], suggesting that the reduction of MTT in the cultures of L929 cells and PBMCs treated with 50% PAM could be considered as non-cytotoxic as well. Our results are in line with previous findings showing that induction of cell death by CAP or PAM treatment is a universal phenomenon in malignant cells [30,31,32,33] in contrast to non-malignant cells, which are more resistant [34]. Towards tumor cells, PAM showed dose- and time-dependent cytotoxic responses in vitro. In addition, the selectivity of PAM against tumor cells is influenced by the medium to be activated and the type of tumor cell lines used [35].

High concentrations of RONS, both originating from PAM and produced by PAM-exposed cancer cells were shown responsible for cell death predominantly via induction of oxidative stress [10,36]. Considering that oxidative stress in dying cells is a hallmark of ICD [37], we analyzed oxidative stress in PAM-treated A375 cells by measuring intracellular ROS with dihydroethidium (DHE) staining (Figure 3b). PAM applied at the concentrations of 25% and higher, induced significant accumulation of ROS in A375 cells after 4 h cultures. Thereby, 100% PAM induced oxidative stress in nearly 80% of cells. After 24 h cultures, even the lower doses of PAM (12.5% PAM) induced significant accumulation of ROS in A375. These results are in line with the general mode of PAM and CAP actions in tumor cells [38]. It is known that ROS production by tumor cells treated with PAM depends on PAM-derived RONS (nitrite and H_2_O_2_) and the generation of initial singlet oxygen (^1^O_2_). This molecule is formed through the complex interaction between NO_2_^−^ and H2O2 and the tumor cells expressing nicotinamide-adenine dinucleotide phosphate oxidase 1 (NOX1), catalase, sodium dismutase (SOD) on their surface [13]. The formation of peroxynitrite (ONOO^−^) plays a key role in these processes [39]. At the site of inactivated catalase, cell-generated H2O2 enters the cell via aquaporins, depletes glutathione, induces the HOCl signaling pathway and promotes lipid peroxidation and cell death by apoptosis [40,41]. Non-malignant cells lack the expression of NOX1, catalase, and SOD on their surface making them resistant to cell death as long as the concentration of H_2_O_2_ is below a cytotoxic threshold level [10]. An investigation showed that the cytoprotective effects of mild PAM against oxidative stress in non-malignant cells such as human skin fibroblasts are characterized by the up-regulation of HO-1 mediated by the Nrf2-ARE pathway [42].

Reactive oxygen species and RNS are the key intracellular signal transducers sustaining autophagy [43]. Autophagy is essential for the survival of cancer cells, since it provides the required energy and removes damaged organelles [44]. However, intensive and persistent activation of autophagy leads to programmed cell death [45]. Moreover, autophagy was shown critical for the induction of the ICD of tumor in a mouse model [46]. Therefore, we next measured autophagy and apoptosis in the A375 treated with PAM for 24 h by quantifying membrane-bound LC3, a key marker of autophagosome formation [47], and by annexin-V/7AAD staining, respectively. It was found that autophagy was indeed triggered in the A375 cells treated with 50% PAM, but not with higher or lower doses of PAM (Figure 3c). According to Annexin V/7AAD staining, both increased apoptosis and necrosis were observed in the A375 cells treated with 50% and 100% PAM for 24 h, whereas lower doses of PAM (25%) induced predominantly the apoptosis of these cells (Figure 3d). Apoptosis was described as a dominant mode of cell death induced by PAM. Adachi et al. [48] showed that PAM reduced the mitochondrial membrane potential, down-regulated the expression of the anti-apoptotic protein Bcl2, activated poly (ADP-ribose) polymerase-1 (PARP-1) and released apoptosis-inducing factor (AIF) from mitochondria, suggesting a caspase-independent apoptotic pathway. Aggressive tumors have a different cellular machinery that protects them from the apoptosis caused by anticancer agents, thereby making them drug resistant. Therefore, cancer therapy based on the induction of non-apoptosis has been considered as an alternative approach to treat apoptosis-resistant cancer cells, including necroptosis [48]. However, the effect of CAP and PAM on this form of cancer cell death has not been sufficiently investigated.

In addition to apoptosis and necrosis, the ICD of tumor cells is characterized by the induction of heat-shock proteins, particularly their plasma membrane localization [49], as well as the release of inflammatory mediators, such as HMBG1, IL-1β and others [50]. In line with this, the membrane expression of heat-shock proteins HSP60 and HSC70 on both A375 and Hep2 cells increased after 4 h treatment with high concentrations of PAM (Figure S1a,c). Moreover, significantly higher levels of IL-1β were detected in 24 h culture supernatants of both tumor cell lines in the presence of 50% and 100% PAM, compared to their levels in the control culture supernatants (Figure S1b,d). Considering that heat-shock proteins and IL-1β are strong stimulators of the immune response [50], these results suggested that PAM-treated tumor cells could strongly potentiate immune response as well.

It has been shown that PAM induced apoptotic cell death in a time-dependent manner in endometrial cancer cells. The results correlated with the G2/M-phase arrest at all PAM concentrations and the induction of intracellular ROS accumulation [51]. In addition, PAM induced autophagy as judged by increased intracellular LC3B protein expression simultaneous with a decrease in the phosphorylated mammalian target of rapamycin (mTOR) and phosphorylated AKT protein levels and a decline of autophagy-related (p62 and ATG family) proteins. The autophagy inhibitor MHY1485 rescued PAM-induced cell death by decreasing the expression of LC3B but without the influence on phosphorylation of mTOR and AKT [51]. Adhikari et al. [52] have recently shown that CAP and synimarin nanoemulsion together activate autophagy in melanoma G-361 cells by activating PI3K/mTOR and EGFR pathways, expressing autophagy-related transcription factors and genes. In contrast, a preliminary report by Ando et al. [53] showed that plasma activated infusion (PAI) solution suppressed the autophagy in melanoma and osteosarcoma cell lines by activating the mTOR pathway. The authors suggested that ROS-mediated necroptosis, but not autophagy, plays a dominant role in the cell death induced by PAI. Our results are in accordance with these, showing that the highest doses of PAM induced predominantly necrosis but not autophagy, and the potentiate induction of ICD markers, such as heat-shock proteins and IL-β. However, all three types (autophagy, apoptosis and necrosis) of cell death are seen with 50% PAM, suggesting that further investigation of these mechanisms is necessary. 

### 3.3. Tumor Lysates Prepared with PAM-Treated Cells Potentiate Maturation of Dendritic Cells

Complete tumor lysates are considered attractive and affordable sources of tumor antigens suitable for an autologous anti-tumor DC vaccine [54]. Several clinical trials used whole autologous tumor lysates prepared by multiple freeze–thaw cycles of tumor cells for DC vaccines [55,56]. Necrotic cell death by freeze-thaw enables the release of DAMPs from tumor cells such as HSP70, HSP90, HMGB1, and others [57], driving the maturation of DCs [58]. However, the finding that freeze-thaw necrotic tumor cells could inhibit toll-like receptor (TLR)-induced maturation and functions of DCs [59], opened serious doubts about the immunogenicity of thus prepared tumor lysates [60]. Different protocols for inducing immunogenic tumor lysates have been described including heat-treatment [61], hydrostatic pressure [62], electroporation [63], and others [60]. Nevertheless, it remained unclear how these protocols compare to standard freeze-thaw tumor lysates, and whether the application of PAM for the treatment of tumor cells could improve the immunogenicity of their lysates. Considering that PAM induced the ICD of A375 cells, we next investigated whether the A375 lysates prepared from A375 cells treated with 100% PAM for 24 h (PAM-A375lys) display better effects on DC maturation compared to the lysates prepared from non-treated A375 cells (A375lys). Thereby, LPS/IFN-γ treatment was additionally used as a strong maturation stimulus potentiating the Th1 polarizing capacity of DCs, which is highly desirable in the DC anti-cancer vaccine [64,65]. 

**Figure 4:**
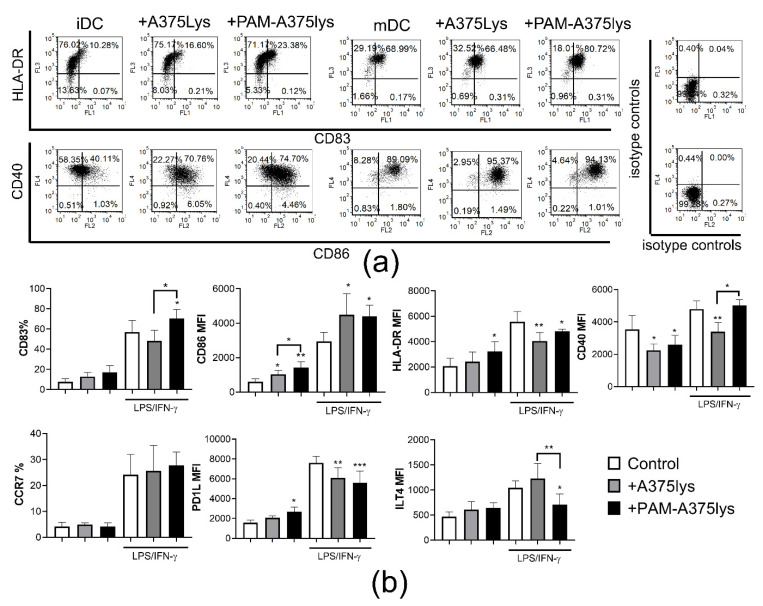
Effects of PAM-treated A375 lysates on phenotypic properties of DCs. Immature DCs differentiated for 4 days in the presence of GM-CSF/IL-4, were treated with the A375 lysate or the PAM-A375 lysate at 1:1 A375: DCs ratio, and after 4 h they were stimulated with LPS/IFN-γ or not, for the next 16 h. (**a**) A representative experiment on CD83/HLA-DR and CD40/CD86 co-expression analysis is shown and, (**b**) the summarized data from 3 independent experiments (different DC donors) are shown for the % of cells expressing the indicated surface marker, or as mean fluorescence intensity (MFI), ± SD. * *p* < 0.05, ** *p* < 0.01, *** *p* < 0.005 vs corresponding control DCs (white bars) or as indicated with lines (RM-ANOVA, Tukey’s multiple comparison test). Statistical significance between the corresponding iDCs and mDCs was not indicated for clarity.

Both A375 lysates increased the expression of the costimulatory CD86 molecule and reduced CD40 expression by immature (im) DC, whereas PAM-A375lys also increased the expression of HLA-DR by imDC compared to control imDC (Figure 4). Upon LPS/IFN-γ treatment, the expression of all markers tested was up-regulated significantly. Thereby, both lysates additionally up-regulated the CD86 expression by mDC and inhibited LPS/IFN-γ-induced up-regulation of HLA-DR and PDL1 by mDC. However, the PAM-treated A375 lysate displayed an additional stimulatory effect on the up-regulation of CD83 on mDC and reduced the LPS/IFN-γ-induced up-regulation of ILT-4 on mDC significantly. In contrast, the control A375 lysate inhibited LPS-induced up-regulation of CD40 on mDC, unlike the PAM-treated A375 lysate. 

Miebach et al. [66] showed recently that colorectal cancer cells treated with an argon-based plasma jet increase the expression of CD80 and CD86 by monocyte-derived DCs in co-culture, unlike a neon-based plasma jet, which induced similar weak (<30%) cytotoxicity in tumor cell culture. However, the majority of CAP-treated tumor cells in the DC co-cultures were live in that study, and it remained unclear how they modulate the maturation and functions of DCs triggered by stimuli. Our study is the first report showing that DCs treated with PAM-tumor lysates display a good immunogenic phenotype, especially after the stimulation with LPS/IFN-γ, according to their higher CD83 and lower expression of ILT-4. ILT-4 was demonstrated as an important new checkpoint molecule in tumor immunotherapy involved in the induction of regulatory T cells [67]. On the other hand, CD83 is critically involved in the maturation of DCs and their resistance to pro-tolerogenic effects of IL-10 [68]. 

### 3.4. Tumor Lysates Prepared from PAM-Treated Cells do Not Impair High IL-12/IL-10 Production Ratio by DCs

**Figure 5:**
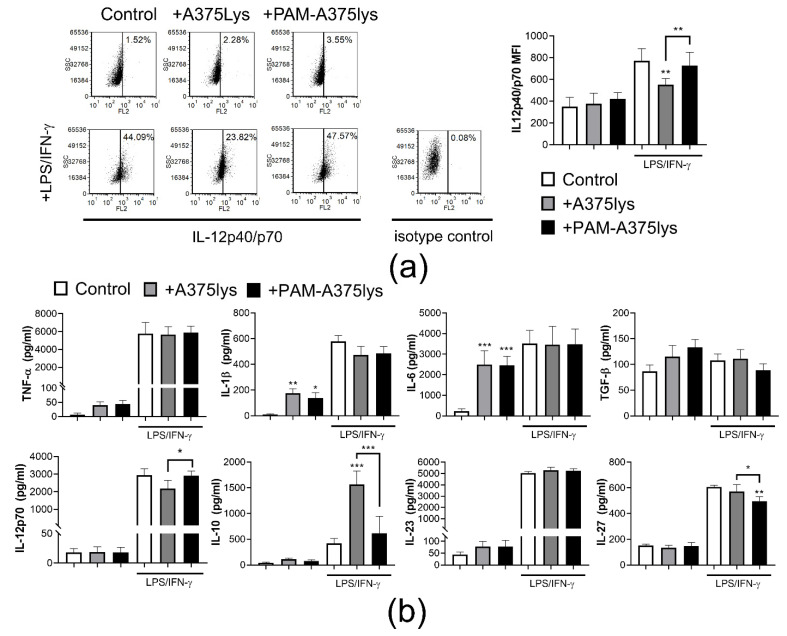
Effects of PAM-treated A375 lysates on cytokines expression by DCs. Immature DCs differentiated for 4 days in the presence of GM-CSF/IL-4, were treated with A375 lysate or PAM-treated A375 lysate at 1:1 A375: DC ratio, and after 4 h they were treated with LPS/IFN-γ or not, for the next 16 h. (**a**) A representative experiment on IL-12p40/p70 expression analysis is shown, and the summarized data from 3 independent experiments (with different DC donors) are shown as mean fluorescence intensity (MFI) ± SD. (**b**) The levels of indicated cytokines detected by ELISA in the cell-culture supernatants are shown as pg/mL ± SD. * *p* < 0.05, ** *p* < 0.01, *** *p* < 0.005 vs. corresponding control DCs (white bars) or as indicated with lines (RM-ANOVA, Tukey’s multiple comparison test). Statistical significance between the corresponding iDCs and mDCs was not indicated for clarity.

Besides the mature phenotype of DCs, the induction of an efficient anti-tumor response by DCs is marked by their increased IL-12 production or IL-12/IL-10 production ratio [69]. Therefore, intracellular expression of IL-12p40/p70 was analyzed in DCs after the cultures with A375 lysates (Figure 5). It was shown that the A375 lysates did not induce significant IL-12 expression in imDCs. The strongest induction of IL-12p40/p70 expression was detected after LPS/IFN-γ treatment of PAM-A375lys-treated mDCs and control mDCs (Figure 5a), whereas mDCs treated with a control A375 lysate displayed significantly lower IL-12p40/p70 expression. 

A similar trend for IL-12 production was observed when protein levels were measured in cell-culture supernatants by ELISA (Figure 5b). However, it was found that control A375lys-treated mDCs produced substantially higher levels of IL-10 compared to PAM-A375lys or control mDCs, suggesting that PAM-A375lys mDCs display a significantly better IL-12/IL-10 production ratio than A375lys-treated mDCs. Previous reports also showed that freeze-thaw tumor lysates stimulate IL-10 production by mouse DCs [59], which could be a consequence of direct inhibitory effects of autocrine IL-10 on the capacity of DCs to produce IL-12 [70]. However, mouse IL-10^−/−^ DCs treated with tumor lysates also displayed a reduced capacity for IL-12 production suggesting that this effect could be independent of IL-10 as well [59]. It remains unclear whether similar mechanisms occur in human DCs treated with tumor lysates, so this requires further investigations. 

PAM-A375lys and control A375lys both increased IL-1β and IL-6 production by imDCs but did not modulate their production by mDCs (Figure 5b). The significance of this finding is still not clear, since a NLRP3-regulated increase in IL-1β, and subsequently IL-6 production was reported to promote tumor growth [71]. In contrast, Ghiringhelli et al. [72] showed that the activation of NLRP3-dependent IL-1β production by DCs, triggered via ATP release from dying tumor cells and activation of the P2X7 purinergic receptor, is critical for the efficient priming of IFN-γ-producing CD8 T cells by dying tumor cells. PAM-A375lys-treated mDCs also produced significantly lower levels of IL-27 compared to both control A375lys-treated mDCs and control mDCs. IL-27 has been implicated in promoting cancer progression [73], and high IL-27 levels are associated with advanced cancer [74], most probably due to its capacity to induce regulatory T cells [75]. Therefore, the attenuating effects of PAM-A375lys on IL-27 production by DCs could be interpreted as a desirable effect for the development of a DC cancer vaccine. 

To observe whether different kinds of tumor cells induce similar effects on DC maturation and IL-12/IL-10 production ratio, Hep2 tumor cells were used instead of A375 to prepare lysates after the PAM treatment. It was observed that mDCs pre-treated with PAM-Hep2lys express higher levels of CD83 and CD86, lower levels of PDL1, and display a higher IL-12/IL-10 production ratio than the control Hep2lys-treated mDCs, although Hep2lys did not stimulate IL-10 production by DCs (Figure S2a,b). PAM-Hep2lys treated mDCs also displayed higher expression of CD86 and lower expression of PD-L1 compared to control mDCs. These results suggest that the phenomenon of increasing the immunogenicity of tumor lysates by PAM is not limited to a single tumor cell line. However, additional investigations are necessary to evaluate whether this phenomenon applies to primary tumors isolated from cancer patients as well. 

We also assessed whether contaminating PAM added to DC cultures along with the lysates (5% PAM) could have directly affected the maturation and the IL-12/IL-10 production ratio by DCs and found no significant effects (data not shown). The cells were also treated with 12.5% PAM, which is more than twice the amount of PAM added with the PAM-A375lys in DC cultures, as well as 25% PAM, as the highest non-toxic dose of PAM for PBMCs (Figure S3). It was found that neither concentration of PAM directly affected oxidative stress and apoptosis in DCs after 24h (Figure S3a,b), nor did it affect the maturation and IL-12 production capacity of DCs (Figure S3c,d). However, 25% of PAM reduced LPS/IFN-γ-induced increase in IL-10 production by these cells. Unlike T and NK cells, which are more susceptible to ROS-inducing treatments [76,77], monocytes and DCs are more resistant due to their stronger anti-oxidative protection systems allowing them to secrete ROS as a part of normal immune functions [12,20,78]. However, an increased presence of exogenous ROS or their prolonged presence could induce depletion of glutathione in DCs leading to their reduced maturation and Th1 polarization capacity [79]. In this sense, 25% PAM induced attenuation of IL-10 production by DCs which could be interpreted as a beneficial effect in tumor therapy. Nevertheless, care should be taken when DCs are exposed to higher doses of PAM or for a longer period, as this could reduce the DC-mediated immune response. Cumulatively, our results suggest that PAM-treated tumor lysates display good stable immunogenic properties on DCs, and that the effect is not merely a direct consequence of the contaminating PAM. 

### 3.5. DCs Loaded with PAM-A375 Lysate Preserve Th1, Potentiate Cytotoxic CD8 T Cells and Th17 Response 

**Figure 6:**
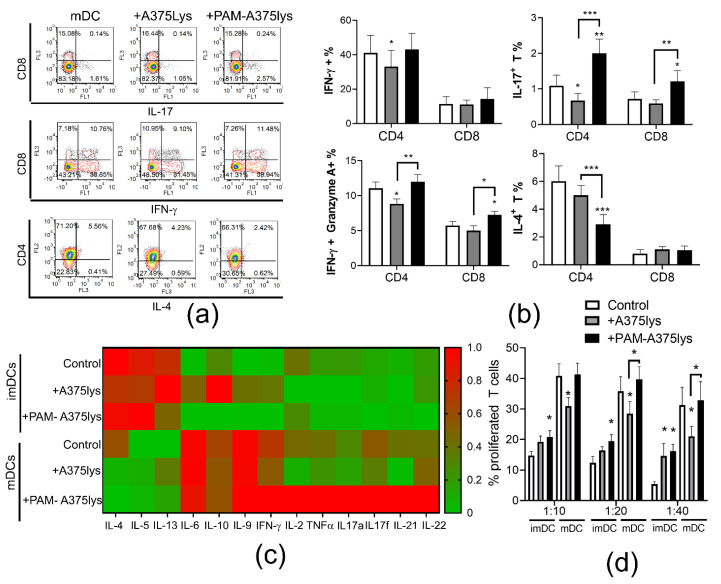
T cell polarization capacity of DCs treated with A375 lysates. (**a**) Immature DCs and mDCs, either treated or not with the A375 lysate (+A375lys) or the PAM-treated A375 lysate (+PAM-A375lys) were co-cultivated with MACS-purified allogeneic T cells (1 × 10^5^/well) at 1:20 (DC: T cell ratio) for 5 days, followed by the stimulation of co-cultures with PMA/Ca Ionophore/monensin for the last 3 h prior to their staining for flow cytometry. Representative dot-plots are shown on the total gated T cells that were co-cultured with the indicated mDCs and afterwards stained to CD4, CD8 and the indicated cytokines. (**b**) The summarized results on the % of cytokine/enzyme expressing cells normalized to 100% of T cells are shown as % ± SD of 3 independent experiments. (**c**) The levels of cytokines from DC/T cell co-cultures as in (**a**), stimulated for the last 3 h with PMA/ Ca ionophore, were measured with the LegendPlex Th13-plex system from the co-culture supernatants. The results are shown as a heatmap wherein each cell represent the level of cytokine normalized in each experiment to the range of 0–1, and averaged from 3 independent experiments, as described in Materials and Methods. (**d**) The allogeneic T cell proliferation was analyzed after 5-day co-cultures of CellTrace Far Red-labeled T cells (1 × 10^5^cell/well) and different number of DC (1 × 10^4^, 0.5 × 10^4^, 0.25 × 10^4^ cells/well), providing 1:10–1:40 DC: T cell ratios. The percentage of proliferated (CellTrace Far Red ^low^) T cells is shown as mean ± SD of 3 independent experiments. * *p* < 0.05, ** *p* < 0.01, *** *p* < 0.005 vs. corresponding control DCs (white bars) or as indicated with lines (RM-ANOVA, Tukey’s multiple comparison test). Statistical significance between the corresponding iDC and mDC was not indicated for clarity.

DC-mediated induction of an efficient anti-tumor response involves their increased capacity to induce Th1 and/or Th17 cells [80]. Therefore, the immunogenic properties of DCs loaded with A375 lysates were next investigated in co-cultures with allogeneic T cells to evaluate their T cell polarization capacity (Figure 6). It was found that PAM-A375lys-treated mDCs significantly increased the proportion of Th17 cells (IL-17A^+^ CD4^+^ T), IL17^+^ CD8 T cells and cytotoxic CD8 (IFN-γ^+^ Granzyme A^+^ CD8^+^) T cells compared to both control mDC and A375lys-treated mDC. According to the unaltered IL-12 production by PAM-A375lys-treated mDCs (Figure 4b), and its role [81], we also did not find altered proportions of Th1 (IFN-γ^+^ CD4^+^T cells) cells in co-culture with these DCs. In contrast, control A375lys-treated DCs reduced the proportion of Th1, Th17 and cytotoxic CD4 T cells (IFN-γ^+^ Granzyme A^+^ CD4^+^) compared to control mDCs, probably due to their increased capacity to secrete IL-10, which is known to inhibit Th1 and Th17 polarization [82]. Moreover, PAM-A375lys-treated mDCs significantly reduced the proportion of Th2 (IL-4^+^ CD4^+^) cells compared to mDCs, unlike A375lys-treated mDCs (Figure 6a,b). In general, mDCs induced lower levels of Th2 cytokines (IL-4, IL-5, IL-13) and higher levels of IL-6, Th9 (IL-9), Th1 (IFN-γ, IL-2, TNF-α) and Th17 (IL17A, IL17F, IL21, IL22) cytokines in DC/T cell co-culture supernatants, as compared to co-cultures with imDCs (Figure 6c). Thereby, PAM-A375lys-treated mDCs were the most potent in relatively lowering the Th2 and increasing the Th1 and Th17 cytokines in co-culture supernatants, confirming that PAM-treated tumor lysates potentiate beneficial anti-tumor properties in DCs. In line with this, Th2 cells demonstrated pro-tumorigenic effects in a mouse tumor model, whereas Th1 and Th17 cells displayed the opposite effects [83,84]. PAM-A375lys-treated mDCs also displayed a significantly higher capacity to stimulate proliferation of allogeneic T cells compared to A375lys-treated mDCs (Figure 6d). These results are in line with the higher levels of IL-2 detected in PAM-A375lys-DC/T co-cultures, better maturation of PAM-A375lys DCs, and their stronger IL-12 production, all of which are critical for the induction of T cell proliferation [85,86,87]. 

Analysis of the alloreactive T cell response in DC co-cultures, does not allow direct assessment of the antigen-specific T cells response. Therefore, in a pilot experiment, we additionally tested whether autologous T cells primed with A375 lysates-loaded DCs proliferate and display cytotoxic activity towards live A375 cells after 6-days of priming with DCs. Encouragingly, we observed that the PAM-A375lys-treated mDCs induced the highest proliferation of autologous T cells in co-cultures (Figure S4a). Additionally, T cells primed with PAM-A375lys-treated mDCs displayed significantly higher cytotoxic activity towards live A375 cells after 4h of co-cultures compared to T cells primed with A375lys-mDCs or control mDCs, when the same number of primed T cells was used in the co-cultures (Figure S4b) suggesting that both increased proliferation and increased cytotoxic functions are potentiated by PAM-A375lys-treated DCs.

The increased proliferation and cytotoxic activity of T cells primed with control DCs which were not treated with tumor lysates, could be attributed to the presence of other proteins during co-cultures such as FCS [88]. Therefore, additional investigations on the antigen-specific effects of DCs, especially with cells from cancer patients, are necessary to delineate the antigen-specific and direct modulatory effects of PAM-A375lys on the DCs capacity to induce proliferation and cytotoxic autologous T cells.

### 3.6. DCs Loaded with PAM-Tumor Lysate Do Not Induce Tregs

**Figure 7:**
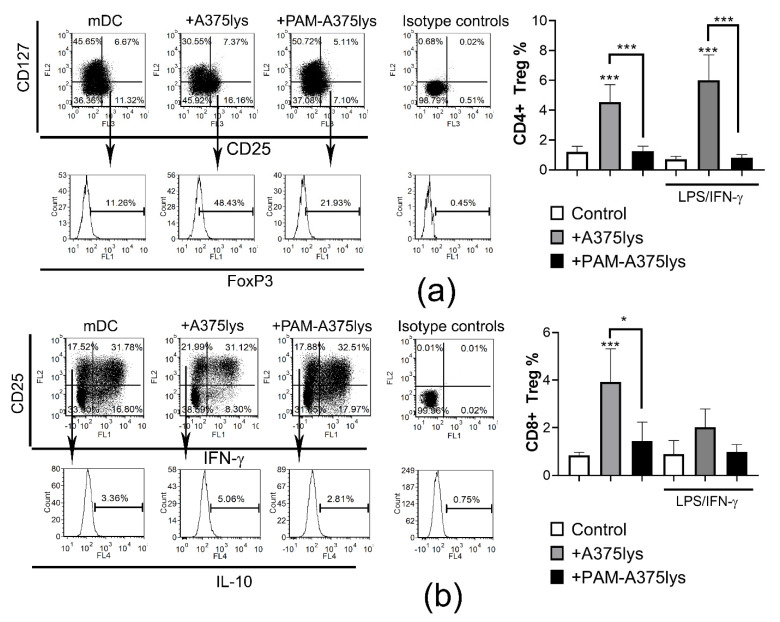
Treg induction capacity of DCs treated with A375 lysates. DCs either treated or not with the A375 lysate (+A375lys) or the PAM-treated A375 lysate (+PAM-A375lys) were co-cultivated with MACS-purified allogeneic T cells (1 × 10^5^/well) at 1:40 (DC: T cell ratio) for 5 days, in the presence of 2 ng/mL of IL-2, followed by the stimulation of the co-cultures with PMA/ Ca Ionophore/ Monensin for the last 3 h prior to their staining for flow cytometry. (**a**) Representative dot-plots are shown on total gated CD4^+^ T cells after staining to CD25, CD127 and intracellular FoxP3. The summarized results on the % of CD4 Tregs are shown as % ± SD of 3 independent experiments. (**b**) Representative dot-plots are shown on total gated CD8^+^ T cells after staining to CD25, and intracellular IFN-γ and IL-10. The summarized results on the % of CD8 Tregs are shown as % ± SD of 3 independent experiments. * *p* < 0.05, *** *p* < 0.005 vs. corresponding control DCs (white bars) or as indicated with lines (RM-ANOVA, Tukey’s multiple comparison test). Statistical significance between the corresponding iDCs and mDCs was not indicated for clarity.

An efficient immunogenic DC vaccine ought to potentiate the Th1/Th17 response in T cells, but not regulatory T cells, which the suppress immune response and display pro-tumorigenic effects [62]. In line with this, previous reports [89], including our own [23,90], have shown that regulatory IL-10-producing CD8^+^ T cells display stronger suppressive activity than conventional FoxP3^+^ CD4^+^ T cells. Therefore, the presence of conventional CD4 Tregs (CD4^+^CD127^−^CD25^+^FoxP3^+^) and regulatory CD8^+^T cells (CD8^+^CD25^+^IL-10^+^IFN-γ^-^) was analyzed in DC/T cell co-cultures. It was found that iDCs treated with control A375 lysates induced significant proportions of regulatory CD4 and CD8 T cells, unlike PAM-A375lys-loaded DCs (Figure 7). Stimulation of A375lys-treated DCs with LPS/IFN-γ did not affect the capacity of these DCs to induce CD4 Tregs, though it did lower their CD8 Treg inducing capacity. The higher capacity of A375lys-treated DCs to induce regulatory T cells is probably a consequence of their higher expression of ILT-4, IL-10, and IL-27 compared to PAM-A375lys-treated DCs. ILT-4 is critical for the induction of CD8^+^CD25^+^IL10^+^ regulatory T cells by DCs, as we showed previously by blocking ILT-4 in the DC/T cell co-cultures [23]. IL-10 was shown to induce both regulatory CD8 and CD4 T cells [89], whereas IL-27 potentiates the functions of CD4^+^ Tregs [91], leading to pro-tumorigenic effects in a multiple gene-deficient mouse model system [92]. Overall, these results suggest that PAM-A375lys induces desirable immunogenic properties in DCs, whereas A375 lysates prepared by the conventional freeze-thaw method could induce adverse effects in DC-vaccines via induction of pro-tumorigenic T cells subsets.

## 4. Conclusions

A DBD atmospheric pressure plasma jet was employed as a CAP source in order to obtain PAM by treating RPMI 1640 culture medium. Three main RONS components (H_2_O_2_, nitrates, and nitrites) were identified in PAM. Tumor cell lines (A375 and Hep2) were more sensitive to the cytotoxic effects of PAM including the stimulation of ROS production, than a non-transformed cell line (L929 cells) and human PBMC. The type of PAM-induced ICD in A375 cells (autophagy, apoptosis, necrosis, membrane expression of heat-shock proteins, and the secretion of IL-1β) depended on applied concentrations of PAM. PAM-A375 lysate potentiated the maturation of DCs by up-regulating CD83 and CD86 expression, simultaneously with a down-regulation of PDL1, and increased the IL-12/IL-10 production ratio by mature DCs, compared to the control A375 lysate without PAM treatment. Mature DCs treated with the PAM-A375 lysate preserved Th1, potentiated Th17 and down-regulated Th2 responses in co-culture with T cells. In addition, such treated DCs increased the frequency and cytotoxic activity of CD8 T cells, along with a reduction in CD8 Tregs frequency. In contrast to the DCs treated with control tumor lysate, which increased the proportion of conventional FoxP3^+^ CD4 Tregs, the PAM-treated tumor lysate did not potentiate the Treg-inducing capacity of DCs. Cumulatively, our results suggest for the first time that priming of mature DCs with tumor cell lysates prepared by PAM-induced ICD could be explored as a new strategy for the generation of more immunogenic DC-based tumor vaccines. 

## Data Availability

All data is shown in the manuscript and raw data is available from corresponding authors upon reasonable request.

## Supplementary Materials

The following are available online at https://www.mdpi.com/article/10.3390/cancers13071626/s1, Figure S1: Dose dependent effects of PAM on induction of heat shock proteins and IL-1β secretion by tumor cell lines, Figure S2: Effects of PAM-treated Hep2 lysates on phenotype and cytokines production by DCs, Figure S3: Direct effects of PAM on oxidative stress, apoptosis, maturation, and cytokines expression by DCs, Figure S4: Proliferation and cytotoxic activity of T cells primed with DCs.

## References

[1] Laroussi M., Lu X., Keidar M. (2017). Perspective: The physics, diagnostics, and applications of atmospheric pressure low temperature plasma sources used in plasma medicine. J. Appl. Phys..

[2] Lu X., Naidis G.V., Laroussi M., Reuter S., Graves D.B., Ostrikov K. (2016). Reactive species in non-equilibrium atmospheric-pressure plasmas: Generation, transport, and biological effects. Phys. Rep..

[3] Kong M.G., Kroesen G., Morfill G., Nosenko T., Shimizu T., Dijk J.v., Zimmermann J.L. (2009). Plasma medicine: An introductory review. New J. Phys..

[4] Keidar M. (2018). A prospectus on innovations in the plasma treatment of cancer. Phys. Plasmas.

[5] Metelmann H.-R., Seebauer C., Miller V., Fridman A., Bauer G., Graves D.B., Pouvesle J.-M., Rutkowski R., Schuster M., Bekeschus S. (2018). Clinical experience with cold plasma in the treatment of locally advanced head and neck cancer. Clin. Plasma Med..

[6] Vandamme M., Robert E., Pesnel S., Barbosa E., Dozias S., Sobilo J., Lerondel S., Pape A.L., Pouvesle J.-M. (2010). Antitumor Effect of Plasma Treatment on U87 Glioma Xenografts: Preliminary Results. Plasma Process. Polym..

[7] Graves D.B. (2014). Reactive Species from Cold Atmospheric Plasma: Implications for Cancer Therapy. Plasma Process. Polym..

[8] Furuta R., Kurake N., Ishikawa K., Takeda K., Hashizume H., Tanaka H., Kondo H., Sekine M., Hori M. (2017). Intracellular responses to reactive oxygen and nitrogen species, and lipid peroxidation in apoptotic cells cultivated in plasma-activated medium. Plasma Process. Polym..

[9] Hirst A.M., Simms M.S., Mann V.M., Maitland N.J., O’Connell D., Frame F.M. (2015). Low-temperature plasma treatment induces DNA damage leading to necrotic cell death in primary prostate epithelial cells. Br. J. Cancer.

[10] Bauer G. (2018). Signal amplification by tumor cells: Clue to the understanding of the antitumor effects of cold atmospheric plasma and plasma-activated medium. IEEE Trans. Radiat. Plasma Med. Sci..

[11] Khalili M., Daniels L., Lin A., Krebs F.C., Snook A.E., Bekeschus S., Bowne W.B., Miller V. (2019). Non-Thermal Plasma-Induced Immunogenic Cell Death in Cancer: A Topical Review. J. Phys. D Appl. Phys..

[12] Van Loenhout J., Peeters M., Bogaerts A., Smits E., Deben C. (2020). Oxidative Stress-Inducing Anticancer Therapies: Taking a Closer Look at Their Immunomodulating Effects. Antioxidants.

[13] Bauer G., Sersenová D., Graves D.B., Machala Z. (2019). Cold Atmospheric Plasma and Plasma-Activated Medium Trigger RONS-Based Tumor Cell Apoptosis. Sci. Rep..

[14] Mohamed H., Esposito R.A., Kutzler M.A., Wigdahl B., Krebs F.C., Miller V. (2020). Nonthermal plasma as part of a novel strategy for vaccination. Plasma Process. Polym..

[15] Wculek S.K., Cueto F.J., Mujal A.M., Melero I., Krummel M.F., Sancho D. (2020). Dendritic cells in cancer immunology and immunotherapy. Nat. Rev. Immunol..

[16] Schlitzer A., McGovern N., Ginhoux F. (2015). Dendritic cells and monocyte-derived cells: Two complementary and integrated functional systems. Semin. Cell Dev. Biol.

[17] Saxena M., Bhardwaj N. (2018). Re-Emergence of Dendritic Cell Vaccines for Cancer Treatment. Trends Cancer.

[18] Lin A., Truong B., Pappas A., Kirifides L., Oubarri A., Chen S., Lin S., Dobrynin D., Fridman G., Fridman A. (2015). Uniform Nanosecond Pulsed Dielectric Barrier Discharge Plasma Enhances Anti-Tumor Effects by Induction of Immunogenic Cell Death in Tumors and Stimulation of Macrophages. Plasma Process. Polym..

[19] Crittenden M., Kohrt H., Levy R., Jones J., Camphausen K., Dicker A., Demaria S., Formenti S. (2015). Current clinical trials testing combinations of immunotherapy and radiation. Semin. Radiat. Oncol..

[20] Bekeschus S., Kolata J., Winterbourn C., Kramer A., Turner R., Weltmann K.D., Bröker B., Masur K. (2014). Hydrogen peroxide: A central player in physical plasma-induced oxidative stress in human blood cells. Free Radic. Res..

[21] Tanaka H., Mizuno M., Ishikawa K., Nakamura K., Kajiyama H., Kano H., Kikkawa F., Hori M. (2011). Plasma-Activated Medium Selectively Kills Glioblastoma Brain Tumor Cells by Down-Regulating a Survival Signaling Molecule, AKT Kinase. Plasma Med..

[22] Yan D., Nourmohammadi N., Bian K., Murad F., Sherman J.H., Keidar M. (2016). Stabilizing the cold plasma-stimulated medium by regulating medium’s composition. Sci. Rep..

[23] Tomić S., Ilić N., Kokol V., Gruden-Movsesijan A., Mihajlović D., Bekić M., Sofronić-Milosavljević L., Čolić M., Vučević D. (2018). Functionalization-dependent effects of cellulose nanofibrils on tolerogenic mechanisms of human dendritic cells. Int. J. Nanomed..

[24] Khlyustova A., Labay C., Machala Z., Ginebra M.-P., Canal C. (2019). Important parameters in plasma jets for the production of RONS in liquids for plasma medicine: A brief review. Front. Chem. Sci. Eng..

[25] Harley J.C., Suchowerska N., McKenzie D.R. (2020). Cancer treatment with gas plasma and with gas plasma-activated liquid: Positives, potentials and problems of clinical translation. Biophys. Rev..

[26] Van Boxem W., Van der Paal J., Gorbanev Y., Vanuytsel S., Smits E., Dewilde S., Bogaerts A. (2017). Anti-cancer capacity of plasma-treated PBS: Effect of chemical composition on cancer cell cytotoxicity. Sci. Rep..

[27] Canal C., Fontelo R., Hamouda I., Guillem-Marti J., Cvelbar U., Ginebra M.-P. (2017). Plasma-induced selectivity in bone cancer cells death. Free Radic. Biol. Med..

[28] Pitt J.M., Kroemer G., Zitvogel L. (2017). Immunogenic and Non-immunogenic Cell Death in the Tumor Microenvironment. Adv. Exp. Med. Biol.

[29] ISO 10993-5:2009(en) Biological Evaluation of Medical Devices—Part 5: Tests for in Vitro Cytotoxicity. https://www.iso.org/obp/ui#iso:std:iso:10993:-5:ed-3:v1:en.

[30] Utsumi F., Kajiyama H., Nakamura K., Tanaka H., Hori M., Kikkawa F. (2014). Selective cytotoxicity of indirect nonequilibrium atmospheric pressure plasma against ovarian clear-cell carcinoma. Springerplus.

[31] Welz C., Emmert S., Canis M., Becker S., Baumeister P., Shimizu T., Morfill G.E., Harréus U., Zimmermann J.L. (2015). Cold Atmospheric Plasma: A Promising Complementary Therapy for Squamous Head and Neck Cancer. PLoS ONE.

[32] Xiang L., Xu X., Zhang S., Cai D., Dai X. (2018). Cold atmospheric plasma conveys selectivity on triple negative breast cancer cells both in vitro and in vivo. Free Radic. Biol. Med..

[33] Saadati F., Mahdikia H., Abbaszadeh H.-A., Abdollahifar M.-A., Khoramgah M.S., Shokri B. (2018). Comparison of Direct and Indirect cold atmospheric-pressure plasma methods in the B16F10 melanoma cancer cells treatment. Sci. Rep..

[34] Jo A., Joh H.M., Chung T.H., Chung J.W. Anticancer Effects of Plasma-Activated Medium Produced by a Microwave-Excited Atmospheric Pressure Argon Plasma Jet. https://www.hindawi.com/journals/omcl/2020/4205640/.

[35] Biscop E., Lin A., Boxem W.V., Loenhout J.V., Backer J.D., Deben C., Dewilde S., Smits E., Bogaerts A.A. (2019). Influence of Cell Type and Culture Medium on Determining Cancer Selectivity of Cold Atmospheric Plasma Treatment. Cancers.

[36] Graves D.B. (2014). Oxy-nitroso shielding burst model of cold atmospheric plasma therapeutics. Clin. Plasma Med..

[37] Moserova I., Truxova I., Garg A.D., Tomala J., Agostinis P., Cartron P.F., Vosahlikova S., Kovar M., Spisek R., Fucikova J. (2017). Caspase-2 and oxidative stress underlie the immunogenic potential of high hydrostatic pressure-induced cancer cell death. Oncoimmunology.

[38] Turrini E., Laurita R., Stancampiano A., Catanzaro E., Calcabrini C., Maffei F., Gherardi M., Colombo V., Fimognari C. Cold Atmospheric Plasma Induces Apoptosis and Oxidative Stress Pathway Regulation in T-Lymphoblastoid Leukemia Cells. https://www.hindawi.com/journals/omcl/2017/4271065/.

[39] Girard P.-M., Arbabian A., Fleury M., Bauville G., Puech V., Dutreix M., Sousa J.S. (2016). Synergistic Effect of H2O2 and NO2 in Cell Death Induced by Cold Atmospheric He Plasma. Sci. Rep..

[40] Bauer G., Sersenová D., Graves D.B., Machala Z. (2019). Dynamics of Singlet Oxygen-Triggered, RONS-Based Apoptosis Induction after Treatment of Tumor Cells with Cold Atmospheric Plasma or Plasma-Activated Medium. Sci. Rep..

[41] Dai X., Bazaka K., Thompson E.W., Ostrikov K. (2020). (Ken) Cold Atmospheric Plasma: A Promising Controller of Cancer Cell States. Cancers.

[42] Horiba M., Kamiya T., Hara H., Adachi T. (2017). Cytoprotective effects of mild plasma-activated medium against oxidative stress in human skin fibroblasts. Sci. Rep..

[43] Filomeni G., De Zio D., Cecconi F. (2015). Oxidative stress and autophagy: The clash between damage and metabolic needs. Cell Death Differ..

[44] Bhutia S.K., Mukhopadhyay S., Sinha N., Das D.N., Panda P.K., Patra S.K., Maiti T.K., Mandal M., Dent P., Wang X.-Y. (2013). Autophagy: Cancer’s friend or foe?. Adv. Cancer Res..

[45] Fulda S. (2017). Autophagy in Cancer Therapy. Front. Oncol..

[46] Michaud M., Martins I., Sukkurwala A.Q., Adjemian S., Ma Y., Pellegatti P., Shen S., Kepp O., Scoazec M., Mignot G. (2011). Autophagy-dependent anticancer immune responses induced by chemotherapeutic agents in mice. Science.

[47] Klionsky D.J., Abdelmohsen K., Abe A., Abedin M.J., Abeliovich H., Arozena A.A., Adachi H., Adams C.M., Adams P.D., Adeli K. (2016). Guidelines for the use and interpretation of assays for monitoring autophagy (3rd edition). Autophagy.

[48] Adachi T., Tanaka H., Nonomura S., Hara H., Kondo S., Hori M. (2015). Plasma-activated medium induces A549 cell injury via a spiral apoptotic cascade involving the mitochondrial-nuclear network. Free Radic. Biol. Med..

[49] Shevtsov M., Huile G., Multhoff G. (2018). Membrane heat shock protein 70: A theranostic target for cancer therapy. Philos. Trans. R. Soc. Lond. B Biol. Sci..

[50] Fucikova J., Kepp O., Kasikova L., Petroni G., Yamazaki T., Liu P., Zhao L., Spisek R., Kroemer G., Galluzzi L. (2020). Detection of immunogenic cell death and its relevance for cancer therapy. Cell Death Dis..

[51] Yoshikawa N., Liu W., Nakamura K., Yoshida K., Ikeda Y., Tanaka H., Mizuno M., Toyokuni S., Hori M., Kikkawa F. (2020). Plasma-activated medium promotes autophagic cell death along with alteration of the mTOR pathway. Sci. Rep..

[52] Adhikari M., Adhikari B., Ghimire B., Baboota S., Choi E.H. (2020). Cold Atmospheric Plasma and Silymarin Nanoemulsion Activate Autophagy in Human Melanoma Cells. Int. J. Mol. Sci..

[53] Ando T., Suzuki-Karasaki M., Suzuki-Karasaki M., Ichikawa J., Ochiai T., Yoshida Y., Haro H., Suzuki-Karasaki Y. (2020). Synergistic anticancer effect of plasma-activated infusion and salinomycin by targeting autophagy and mitochondrial morphology. bioRxiv.

[54] González F.E., Gleisner A., Falcón-Beas F., Osorio F., López M.N., Salazar-Onfray F. (2014). Tumor cell lysates as immunogenic sources for cancer vaccine design. Hum. Vaccin Immunother..

[55] Nakai N., Hartmann G., Kishimoto S., Katoh N. (2010). Dendritic cell vaccination in human melanoma: Relationships between clinical effects and vaccine parameters. Pigment. Cell Melanoma. Res..

[56] Draube A., Klein-González N., Mattheus S., Brillant C., Hellmich M., Engert A., von Bergwelt-Baildon M. (2011). Dendritic cell based tumor vaccination in prostate and renal cell cancer: A systematic review and meta-analysis. PLoS ONE.

[57] Hernández-Granados A.J., Franco-Molina M.A., Coronado-Cerda E.E., Zapata-Benavides P., Gamboa E.M., Ramos-Zayas Y., Santana-Krímskaya S.E., Rodríguez-Padilla C. (2018). Immunogenic potential of three transmissible venereal tumor cell lysates to prime canine-dendritic cells for cancer immunotherapy. Res. Vet. Sci..

[58] Nace G., Evankovich J., Eid R., Tsung A. (2012). Dendritic Cells and Damage-Associated Molecular Patterns: Endogenous Danger Signals Linking Innate and Adaptive Immunity. J. Innate. Immun..

[59] Hatfield P., Merrick A.E., West E., O’Donnell D., Selby P., Vile R., Melcher A.A. (2008). Optimization of dendritic cell loading with tumor cell lysates for cancer immunotherapy. J. Immunother..

[60] Vandenberk L., Belmans J., Van Woensel M., Riva M., Van Gool S.W. (2016). Exploiting the Immunogenic Potential of Cancer Cells for Improved Dendritic Cell Vaccines. Front. Immunol..

[61] Rojas-Sepúlveda D., Tittarelli A., Gleisner M.A., Ávalos I., Pereda C., Gallegos I., González F.E., López M.N., Butte J.M., Roa J.C. (2018). Tumor lysate-based vaccines: On the road to immunotherapy for gallbladder cancer. Cancer Immunol. Immunother..

[62] Hradilova N., Sadilkova L., Palata O., Mysikova D., Mrazkova H., Lischke R., Spisek R., Adkins I. (2017). Generation of dendritic cell-based vaccine using high hydrostatic pressure for non-small cell lung cancer immunotherapy. PLoS ONE.

[63] Kamigaki T., Kaneko T., Naitoh K., Takahara M., Kondo T., Ibe H., Matsuda E., Maekawa R., Goto S. (2013). Immunotherapy of autologous tumor lysate-loaded dendritic cell vaccines by a closed-flow electroporation system for solid tumors. Anticancer Res..

[64] Tomić S., Janjetović K., Mihajlović D., Milenković M., Kravić-Stevović T., Marković Z., Todorović-Marković B., Spitalsky Z., Micusik M., Vučević D. (2017). Graphene quantum dots suppress proinflammatory T cell responses via autophagy-dependent induction of tolerogenic dendritic cells. Biomaterials.

[65] Jin P., Han T.H., Ren J., Saunders S., Wang E., Marincola F.M., Stroncek D.F. (2010). Molecular signatures of maturing dendritic cells: Implications for testing the quality of dendritic cell therapies. J. Transl. Med..

[66] Miebach L., Freund E., Horn S., Niessner F., Sagwal S.K., von Woedtke T., Emmert S., Weltmann K.-D., Clemen R., Schmidt A. (2021). Tumor cytotoxicity and immunogenicity of a novel V-jet neon plasma source compared to the kINPen. Sci. Rep..

[67] Gao A., Sun Y., Peng G. (2018). ILT4 functions as a potential checkpoint molecule for tumor immunotherapy. Biochim. Biophys. Acta (BBA) Rev. Cancer.

[68] Tze L.E., Horikawa K., Domaschenz H., Howard D.R., Roots C.M., Rigby R.J., Way D.A., Ohmura-Hoshino M., Ishido S., Andoniou C.E. (2011). CD83 increases MHC II and CD86 on dendritic cells by opposing IL-10-driven MARCH1-mediated ubiquitination and degradation. J. Exp. Med..

[69] Castiello L., Sabatino M., Ren J., Terabe M., Khuu H., Wood L.V., Berzofsky J.A., Stroncek D.F. (2017). Expression of CD14, IL10, and Tolerogenic Signature in Dendritic Cells Inversely Correlate with Clinical and Immunologic Response to TARP Vaccination in Prostate Cancer Patients. Clin. Cancer Res..

[70] Loscher C.E., Draper E., Leavy O., Kelleher D., Mills K.H.G., Roche H.M. (2005). Conjugated linoleic acid suppresses NF-kappa B activation and IL-12 production in dendritic cells through ERK-mediated IL-10 induction. J. Immunol..

[71] Lee H.E., Lee J.Y., Yang G., Kang H.C., Cho Y.-Y., Lee H.S., Lee J.Y. (2019). Inhibition of NLRP3 inflammasome in tumor microenvironment leads to suppression of metastatic potential of cancer cells. Sci. Rep..

[72] Ghiringhelli F., Apetoh L., Tesniere A., Aymeric L., Ma Y., Ortiz C., Vermaelen K., Panaretakis T., Mignot G., Ullrich E. (2009). Activation of the NLRP3 inflammasome in dendritic cells induces IL-1β–dependent adaptive immunity against tumors. Nat. Med..

[73] Jia H., Dilger P., Bird C., Wadhwa M. (2016). IL-27 Promotes Proliferation of Human Leukemic Cell Lines Through the MAPK/ERK Signaling Pathway and Suppresses Sensitivity to Chemotherapeutic Drugs. J. Interferon. Cytokine Res..

[74] Diakowska D., Lewandowski A., Markocka-Mączka K., Grabowski K. (2013). Concentration of serum interleukin-27 increase in patients with lymph node metastatic gastroesophageal cancer. Adv. Clin. Exp. Med..

[75] Hall A.O., Beiting D.P., Tato C., John B., Oldenhove G., Lombana C.G., Pritchard G.H., Silver J.S., Bouladoux N., Stumhofer J.S. (2012). The cytokines interleukin 27 and interferon-γ promote distinct Treg cell populations required to limit infection-induced pathology. Immunity.

[76] Takahashi A., Hanson M.G.V., Norell H.R., Havelka A.M., Kono K., Malmberg K.-J., Kiessling R.V.R. (2005). Preferential cell death of CD8+ effector memory (CCR7-CD45RA-) T cells by hydrogen peroxide-induced oxidative stress. J. Immunol..

[77] Mimura K., Kua L.-F., Shimasaki N., Shiraishi K., Nakajima S., Siang L.K., Shabbir A., So J., Yong W.-P., Kono K. (2017). Upregulation of thioredoxin-1 in activated human NK cells confers increased tolerance to oxidative stress. Cancer Immunol. Immunother..

[78] Seres T., Knickelbein R.G., Warshaw J.B., Johnston R.B. (2000). The phagocytosis-associated respiratory burst in human monocytes is associated with increased uptake of glutathione. J. Immunol..

[79] Kim H.-J., Barajas B., Chan R.C.-F., Nel A.E. (2007). Glutathione depletion inhibits dendritic cell maturation and delayed-type hypersensitivity: Implications for systemic disease and immunosenescence. J. Allergy Clin. Immunol..

[80] Yamamoto M., Kamigaki T., Yamashita K., Hori Y., Hasegawa H., Kuroda D., Moriyama H., Nagata M., Ku Y., Kuroda Y. (2009). Enhancement of anti-tumor immunity by high levels of Th1 and Th17 with a combination of dendritic cell fusion hybrids and regulatory T cell depletion in pancreatic cancer. Oncol. Rep..

[81] Tsung K., Meko J.B., Peplinski G.R., Tsung Y.L., Norton J.A. (1997). IL-12 induces T helper 1-directed antitumor response. J. Immunol..

[82] Su J., Chen T., Ji X.-Y., Liu C., Yadav P.K., Wu R., Yang P., Liu Z. (2013). IL-25 Downregulates Th1/Th17 Immune Response in an IL-10–Dependent Manner in Inflammatory Bowel Disease. Inflamm. Bowel Dis..

[83] Ziegler A., Heidenreich R., Braumüller H., Wolburg H., Weidemann S., Mocikat R., Röcken M. (2009). EpCAM, a human tumor-associated antigen promotes Th2 development and tumor immune evasion. Blood.

[84] Muranski P., Boni A., Antony P.A., Cassard L., Irvine K.R., Kaiser A., Paulos C.M., Palmer D.C., Touloukian C.E., Ptak K. (2008). Tumor-specific Th17-polarized cells eradicate large established melanoma. Blood.

[85] Soong R.-S., Song L., Trieu J., Lee S.Y., He L., Tsai Y.-C., Wu T.-C., Hung C.-F. (2014). Direct T Cell Activation via CD40 Ligand Generates High Avidity CD8+ T Cells Capable of Breaking Immunological Tolerance for the Control of Tumors. PLoS ONE.

[86] Henry C.J., Ornelles D.A., Mitchell L.M., Brzoza-Lewis K.L., Hiltbold E.M. (2008). IL-12 Produced by Dendritic Cells Augments CD8+ T cell Activation through the Production of the Chemokines CCL1 and CCL17. J. Immunol..

[87] Wuest S.C., Edwan J., Martin J.F., Han S., Perry J.S.A., Cartagena C.M., Matsuura E., Maric D., Waldmann T.A., Bielekova B. (2011). A vital role for IL-2 trans-presentation in DC-mediated T cell activation in humans as revealed by daclizumab therapy. Nat. Med..

[88] Pullen A.M., Munro A.J., Atassi M.Z. (1987). “Auto-Reactive” T-Cell Hybridomas and the Role of Foetal Calf Serum. Immunobiology of Proteins and Peptides IV: T-Cell Recognition and Antigen Presentation.

[89] Xu Z., Ho S., Chang C.-C., Zhang Q.-Y., Vasilescu E.-R., Vlad G., Suciu-Foca N. (2016). Molecular and Cellular Characterization of Human CD8 T Suppressor Cells. Front. Immunol..

[90] Tomić S., Joksimović B., Bekić M., Vasiljević M., Milanović M., Čolić M., Vučević D. (2019). Prostaglanin-E2 Potentiates the Suppressive Functions of Human Mononuclear Myeloid-Derived Suppressor Cells and Increases Their Capacity to Expand IL-10-Producing Regulatory T Cell Subsets. Front. Immunol..

[91] Nguyen Q.T., Jang E., Le H.T., Kim S., Kim D., Dvorina N., Aronica M.A., Baldwin W.M., Asosingh K., Comhair S. (2019). IL-27 targets Foxp3^+^ Tregs to mediate antiinflammatory functions during experimental allergic airway inflammation. JCI Insight.

[92] Park Y.-J., Ryu H., Choi G., Kim B.-S., Hwang E.S., Kim H.S., Chung Y. (2019). IL-27 confers a protumorigenic activity of regulatory T cells via CD39. Proc. Natl. Acad. Sci. USA.

