# Supplementary material for: Plasma-Activated Medium Potentiates the Immunogenicity of Tumor Cell Lysates for Dendritic Cell-Based Cancer Vaccines
Source: Cancers (Basel). 2021 Apr 1;13(7):1626. doi: 10.3390/cancers13071626 (PMC8037863; doi:10.3390/cancers13071626)
Supplement: Supplementary file 1 [file cancers-13-01626-s001.pdf]

Supplementary Materials: Plasma-Activated Medium Potentiates the Immunogenicity of Tumor Cell Lysates for Dendritic Cell-Based Cancer Vaccines

Sergej Tomić, Anđelija Petrović, Nevena Puač, Nikola Škoro, Marina Bekić, Zoran Petrović and Miodrag Čolić

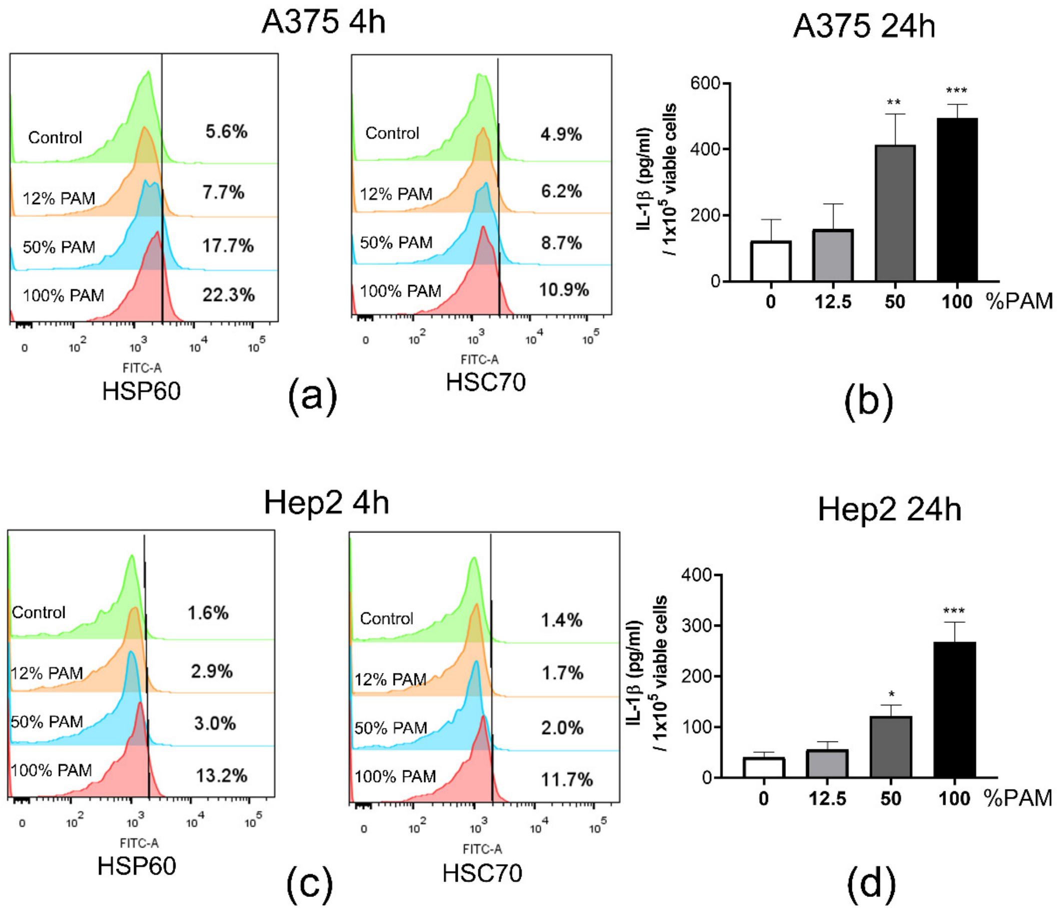

Figure S1. Dose dependent effects of PAM on induction of heat shock proteins and IL-1β secretion by tumor cell lines. Membrane expression of HSP60 and HSC70 by (**a**) A375 and (**c**) Hep2 cells, was analyzed by flow cytometry after 4 h of cultivation of 1×10^5 cells with different dilutions of PAM (0% (control), 12.5%, 50% and 100% in complete RPMI medium, after the staining of non-permeabilized cells with anti-HSP60 and anti-HSC70 primary antibodies followed by secondary anti-IgG Ab. Gate (vertical line) was set according to the puled cells samples stained with secondary antibody only (i.e., without primary antibodies). Representative data out of two experiments with similar results are shown. The levels of IL-1β in supernatants of (**b**) A375 and (**d**) Hep2 cultures carried out with indicated doses of PAM for 24 h are shown as the relative concentration (pg/ml) of IL-1β normalized to the same number (1×10^5) of viable cells. The viability of the cells after 24h cultures was calculated according to the number of seeded cells and the results from MTT assay (Figure 3). Data from two independent experiments, each carried out in triplicates, are shown as mean ± SD. * $p < 0.05$, ** $p < 0.01$, *** $p < 0.005$ compared to non-treated control cells (0% PAM).

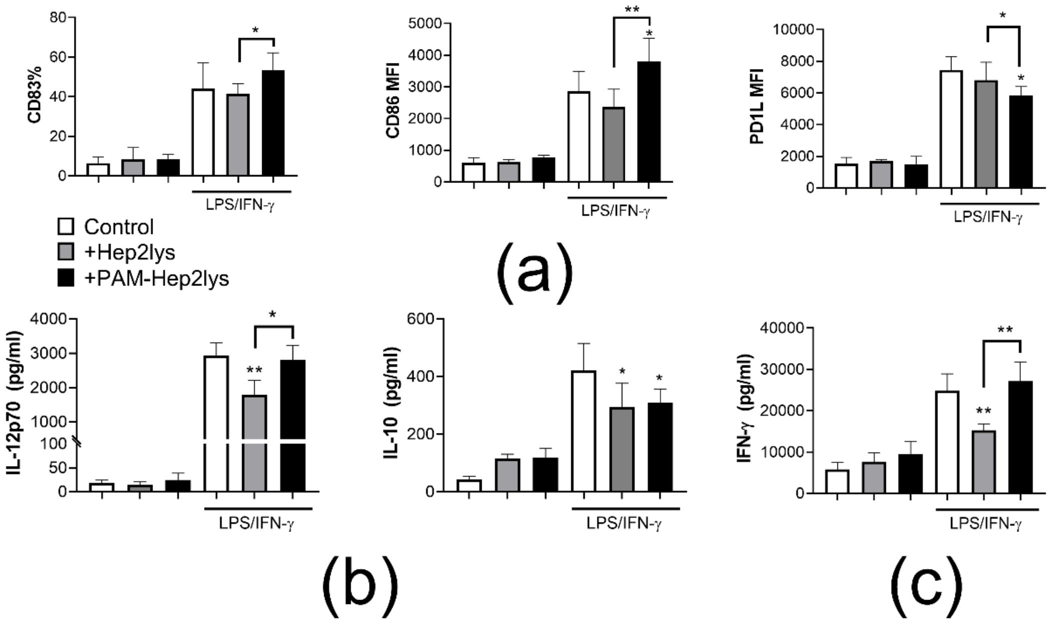

Figure S2. Effects of PAM-treated Hep2 lysates on phenotype and cytokines production by DCs. imDCs, differentiated for 4 days in the presence of GM-CSF/IL-4, were treated with PAM-treated and untreated Hep2 cell lysate at 1:1 Hep2 : DC ratio, and after 4h treated with LPS/IFN-γ or not, for the next 16h. (**a**) Summarized data on CD83, CD86 and PDL1 expression from 3 independent experiments are shown for the % of cells expressing the marker, or as mean fluorescence intensity (MFI), ± SD. (**b**) Levels of IL-12 and IL-10 were detected by ELISA in cell-culture supernatants and the results are shown as pg/ml ± SD from 3 independent experiments. (**c**) The levels of IFN-γ in allogeneic T cell/DCs co-cultures carried out for 5 days, and stimulated for the last 3 h with PMA/ Ca ionophore, were measured with LegendPlex Th13-plex system from the co-culture supernatants. Data from 3 independent experiments are shown as mean pg/ml ± SD. * $p < 0.05$, ** $p < 0.01$, vs corresponding control DCs or as indicated by lines (RM-ANOVA, Tukey's multiple comparison test). Statistical significance between corresponding iDCs and mDCs was not indicated for clarity.

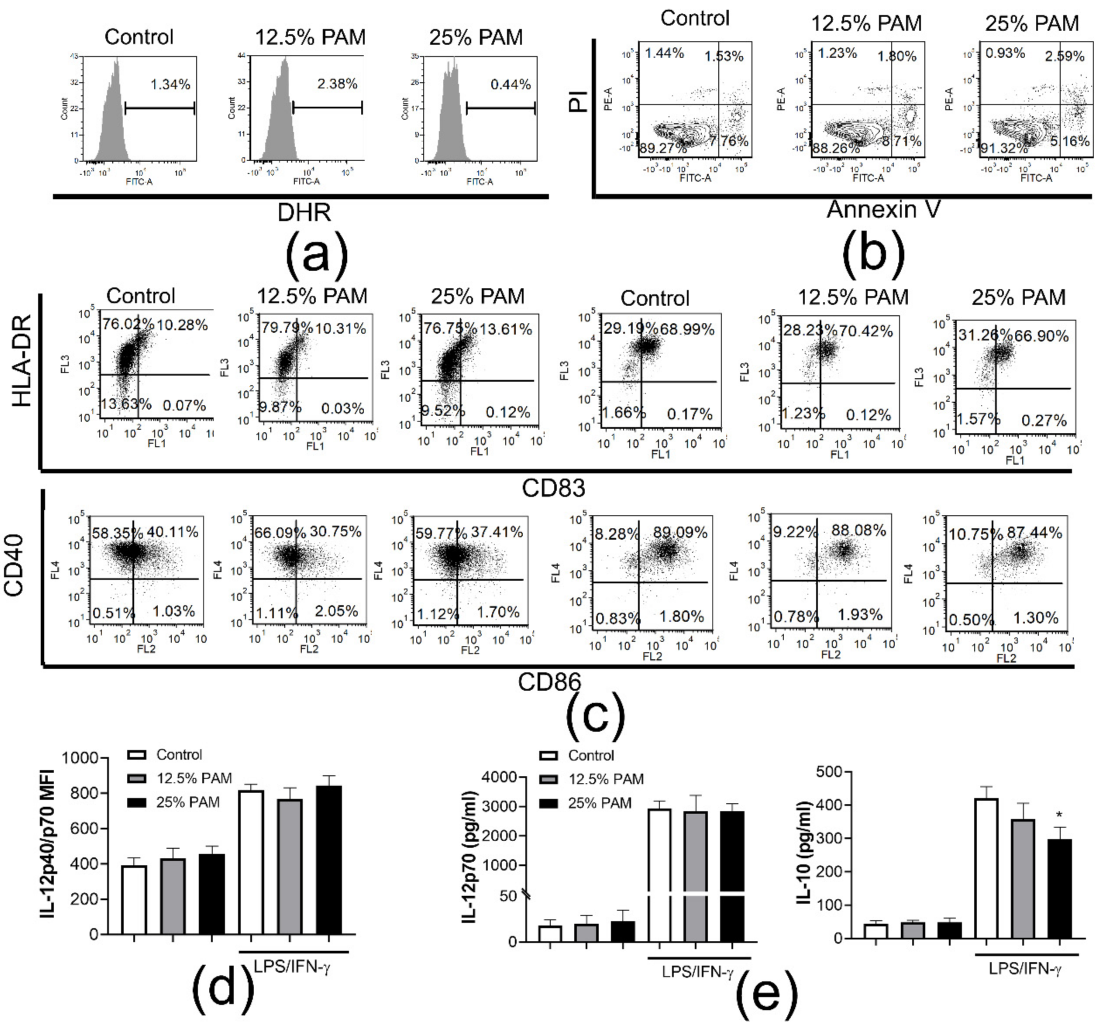

Figure S3. Direct effects of PAM on oxidative stress, apoptosis, maturation, and cytokines expression by DCs. (**a**) Immature DC, differentiated for 4 days in the presence of GM-CSF/IL-4, were treated with 12.5% or 50% PAM and after 24h they were stained for 30 minutes with dihydrorhodamine (DHR) or (**b**) Annexin-V-FITC/PI. A representative experiment is shown out of two with similar results. (**c**) iDCs differentiated as in (**a**) were treated with LPS/IFN-γ or not, for the next 16h followed by phenotype analysis of CDD83/HLA-DR and CD86/CD40 co-expression by flow cytometry. (**d**) Summarized data on the expression levels of IL-12p40/p70 by DCs treated as in (a) are shown as MFI ± SD. (**e**) Levels of IL-12p70, and IL-10 were detected by ELISA in DCs cell-culture supernatants are shown as pg/ml. * $p < 0.05$ vs corresponding control DCs (white bars) or as indicated with lines (RM-ANOVA, Tukey's multiple comparison test). Statistical significance between corresponding iDCs and mDCs was not indicated for clarity.

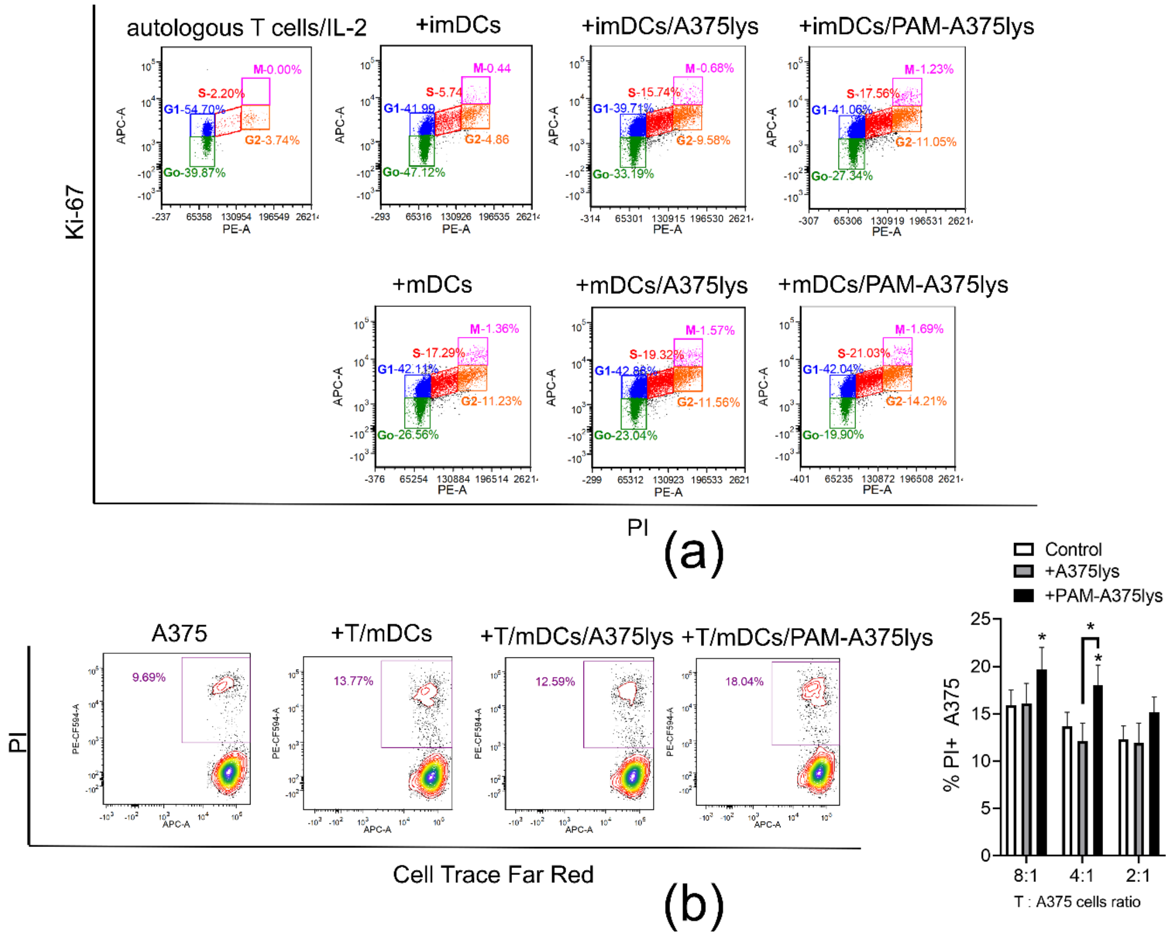

Figure S4. Proliferation and cytotoxic activity of T cells primed with DCs. iDCs and mDCs, either treated or not with A375 lysate (A375lys) or PAM-treated A375 lysate (PAM-A375lys) were co-cultivated with MACS-purified autologous T cells ($1x10^5$/well) at 1:40 (DC: T cell ratio) for 6 days in the presence of IL-2 (10 ng/ml). After that (**a**) the proliferation of autologous T cells was assessed by Ki-67/PI staining of ethanol permeabilized cells, allowing the identification of T cells in G0 (Ki-67^-), G1, S, G2 (Ki-67^{low}) or M (Ki-67^{hi}) phase of cell cycle. A representative experiment, out of two with similar results, is shown. (**b**) The analysis of Cell Trace Far Red labeled-A375 cell death (PI + cells) ($5x10^5$ cells) is shown, after 4h co-cultures with T cells (at 1:2, 1:4 and 1:8 A375: T cell ratio) that were previously primed as in (**a**), either as a representative dot-plots (from 1:4 A375:T cell ratio) or summarized data from three independent experiments as mean ± SD. * $p < 0.05$ compared to control mDCs, or as indicated by line. (RM-ANOVA, Tukey's multiple comparison test).

© 2021 by the authors. Licensee MDPI, Basel, Switzerland. This article is an open access article distributed under the terms and conditions of the Creative Commons Attribution (CC BY) license (http://creativecommons.org/licenses/by/4.0/).
